# Single-cell RNA sequencing of human lung innate lymphoid cells in the vascular and tissue niche reveals molecular features of tissue adaptation

**DOI:** 10.1093/discim/kyad007

**Published:** 2023-06-24

**Authors:** Arlisa Alisjahbana, Imran Mohammad, Yu Gao, Elza Evren, Tim Willinger

**Affiliations:** Department of Medicine Huddinge, Center for Infectious Medicine, Karolinska Institutet, Karolinska University Hospital, Stockholm, Sweden; Department of Medicine Huddinge, Center for Infectious Medicine, Karolinska Institutet, Karolinska University Hospital, Stockholm, Sweden; Department of Otolaryngology-Head and Neck Surgery, Stanford Cancer Institute, Institute for Stem Cell Biology and Regenerative Medicine, Stanford University School of Medicine, Stanford, CA, USA; Department of Medicine Huddinge, Center for Infectious Medicine, Karolinska Institutet, Karolinska University Hospital, Stockholm, Sweden; Department of Medicine Huddinge, Center for Infectious Medicine, Karolinska Institutet, Karolinska University Hospital, Stockholm, Sweden; Comprehensive Pneumology Center (CPC) with the CPC-M bioArchive/Institute of Lung Health and Immunity (LHI), Helmholtz Zentrum München; Member of the German Center for Lung Research (DZL), Munich, Germany; Department of Medicine Huddinge, Center for Infectious Medicine, Karolinska Institutet, Karolinska University Hospital, Stockholm, Sweden

**Keywords:** innate lymphoid cells, lung, niche, tissue residency, humanized mice, single-cell RNA sequencing

## Abstract

Innate lymphoid cells (ILCs) are sentinels of healthy organ function, yet it is unknown how ILCs adapt to distinct anatomical niches within tissues. Here, we used a unique humanized mouse model, MISTRG mice transplanted with human hematopoietic stem and progenitor cells (HSPCs), to define the gene signatures of human ILCs in the vascular versus the tissue (extravascular) compartment of the lung. Single-cell RNA sequencing in combination with intravascular cell labeling demonstrated that heterogeneous populations of human ILCs and natural killer (NK) cells occupied the vascular and tissue niches in the lung of HSPC-engrafted MISTRG mice. Moreover, we discovered that niche-specific cues shape the molecular programs of human ILCs in the distinct sub-anatomical compartments of the lung. Specifically, extravasation of ILCs into the lung tissue was associated with the upregulation of genes involved in the acquisition of tissue residency, cell positioning within the lung, sensing of tissue-derived signals, cellular stress responses, nutrient uptake, and interaction with other tissue-resident immune cells. We also defined a core tissue signature shared between human ILCs and NK cells in the extravascular space of the lung, consistent with imprinting by signals from the local microenvironment. The molecular characterization of human ILCs and NK cells in the vascular and tissue niches of the lung provides new knowledge on the mechanisms of ILC tissue adaptation and represents a resource for further studies.

## Graphical Abstract

**Graphical Abstract:**
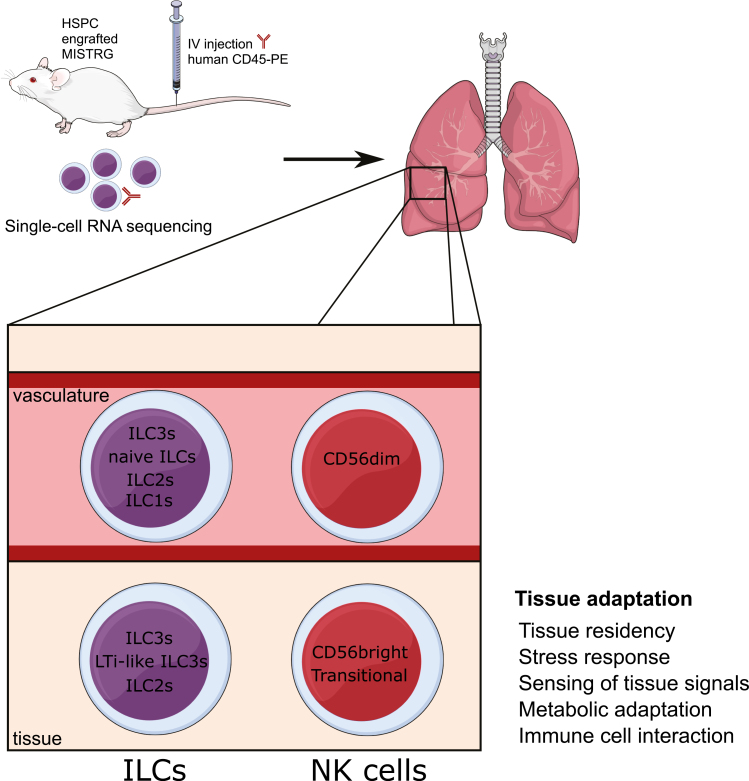

## Introduction

Innate lymphoid cells (ILCs) are innate immune cells that perform important functions such as surveying tissues, preserving organ homeostasis and participating in barrier immunity [1]. Accordingly, ILCs are present in many tissues, including the lung. Mouse studies have established the notion that ILCs are mainly tissue-resident cells, at least in a steady state [2]. Like other tissue-resident immune cells, such as T lymphocytes [3–5], ILCs respond to signals from their surrounding microenvironment [6–10]. However, how human ILCs take up residence in the lung and how they are shaped by anatomically defined tissue niches is poorly understood.

The ILC family includes cytotoxic natural killer (NK) cells and helper-like ILCs. In analogy to T cells, helper-like ILCs are grouped into distinct subsets based on the expression of transcription factors and signature cytokines [1]. ILCs have been extensively studied in mice, but there are relevant differences in ILCs between mice and humans [10, 11]. For example, ILC2s outnumber other lung ILC subset in mice [12, 13], whereas in the human lung, ILC3s are predominant [7, 14]. ILCs have been characterized in human tissues, but studies of human ILCs are largely limited to experiments *ex vivo*.

Mice with a human immune system offer the opportunity to overcome the challenge of studying human ILCs *in vivo* [11, 15]. For this purpose, we have used the MISTRG humanized mouse model that provides human factors through gene knock-in [16]. The provision of human cytokines (M-CSF, IL-3, GM-CSF, TPO) and factors promoting phagocytic tolerance (SIRPα) creates a highly permissive environment for human hematopoiesis in immunodeficient *Rag2*^−/−^*Il2rg*^−/−^ mice. Accordingly, MISTRG mice support the reconstitution of a human innate immune system, including NK cells and ILCs, after transplantation with human CD34^+^ human hematopoietic stem and progenitor cells (HSPCs) [17–19]. We recently showed that human ILCs are present within the vascular and tissue niche of the lung in MISTRG mice engrafted with human CD34^+^ HSPCs [18]. However, the molecular mechanisms underlying the tissue adaptation of human ILCs in the lung are unknown.

Here, we visualized at the single-cell resolution the compartmentalization of human lung ILCs and their associated transcriptional programs in the lung vasculature versus the extravascular space. We found that distinct human ILC populations were distributed between the vascular and tissue niche of the lung. Human ILCs within lung tissue preferentially expressed genes regulating tissue residency, immune cell communication, sensing of tissue status, metabolic adaptation, and response to environmental stress. Our findings represent a valuable resource for future studies on human ILCs and could point towards molecular targets to modify ILC function in human disease.

## Results

### Visualizing vascular and tissue ILCs in the lung of HSPC-engrafted MISTRG mice

MISTRG mice transplanted with human CD34^+^ HSPCs support the development of all types of human ILCs and NK cells in various organs, including the lung [17–19]. Flow cytometry demonstrated that there were significantly more human ILC3s than ILC1s and ILC2s in the lung of HSPC-engrafted MISTRG mice (Supplementary Fig. S1A and B) and the frequency of human ILC subsets in the lung of HSPC-engrafted MISTRG mice was similar to what has been reported for the human lung [7, 14].

To visualize human ILCs in the intravascular and the extravascular compartment of the lung, we performed intravascular cell labeling by the intravenous (IV) injection of a phycoerythrin (PE)-conjugated anti-human CD45 antibody (Supplementary Fig. S1C) as in our previous studies [17, 18, 20]. This antibody binds to blood-exposed human CD45^+^ hematopoietic cells, including ILCs and NK cells. We carefully titrated the anti-human CD45-PE antibody *in vivo* to ensure optimal binding of the antibody to human CD45^+^ cells in the circulation without any excess free antibody (Supplementary Fig. S1D). We previously showed that the IV-injected antibody does not label human CD45^+^ cells in the airways of HSPC-engrafted MISTRG mice [17, 20], demonstrating that no leakage of the antibody into the extravascular space occurred. As further validation, we examined expression of the tissue residency marker CD103 on human ILCs and NK cells in HSPC-engrafted MISTRG mice. We found that CD103^+^ ILCs and NK cells in the lung, which are known to be tissue-resident cells, were not labeled by the IV CD45-PE antibody (Supplementary Figs. S1E and F), further confirming the validity of our intravascular cell labeling approach. Finally, we observed that the frequency of CD69^+^ ILC3s was highest in the lung tissue but also higher in the lung vasculature than in the blood (Supplementary Fig. S1F). A similar pattern was observed for ILC2s, although the increase in CD69^+^ intravascular ILC2s did not reach statistical significance. In contrast, intravascular lung ILC1s and NK cells did not show a higher frequency of CD69^+^ cells than ILC1s and NK cells in the blood (Supplementary Fig. S1F). The stepwise upregulation of CD69 inferred that ILC3s (and possibly ILC2s) migrating from the blood into the lung already start acquiring a tissue residency phenotype in the local vasculature prior to their entry into the lung tissue. It also implied that IV-CD45-PE^+^ ILC3s in the lung are not simply recirculating but are likely adhering to the lung endothelium. Overall, these findings suggest that ILC3s (and possibly ILC2s) in the intravascular compartment of the lung are distinct from their counterparts in the systemic circulation.

### Heterogeneity of human lung ILCs in the vascular and tissue niche

**Figure 1:**
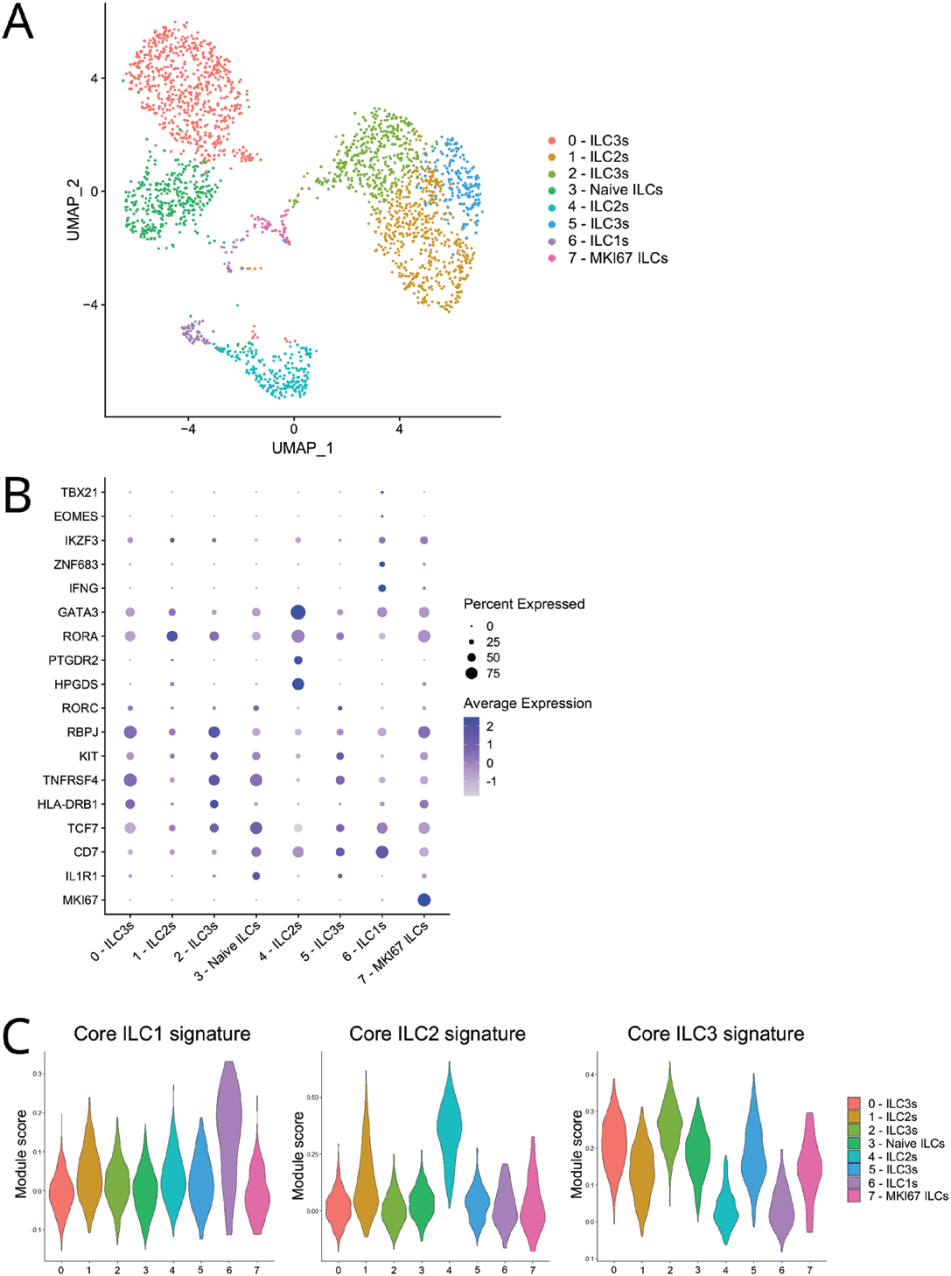
heterogeneity of human ILCs in the lung of HSPC-engrafted MISTRG mice. (A) UMAP of human ILCs clusters from the lung of HSPC-engrafted MISTRG mice as identified by single-cell RNA sequencing. The UMAP consists of 2539 cells. (B) Dot plots showing expression of selected ILC signature genes in the human lung ILC clusters. (**C)** Gene module score analysis to examine the similarity of the ILC clusters from (A) to human ILC1s, ILC2s, and ILC3s in terms of their gene signatures. Core ILC1, ILC2, and ILC3 signatures were obtained from [7], see Supplementary Table S2. Data are from one single-cell RNA-sequencing experiment with pooled lung cells from 10 MISTRG mice that were transplanted with human CD34^+^ HSPCs from different donors.

We next combined intravascular cell labeling with single-cell RNA sequencing to define the gene signatures of vascular and tissue ILCs in the lung of HSPC-engrafted MISTRG mice. For this purpose, ILCs were purified as human CD45^+^CD127^+^CD94^−^ cells lacking T cell and other lineage (Lin) markers and divided into intravascular (IV-CD45-PE^+^) and extravascular (IV-CD45-PE^−^) subsets. Single-cell RNA-sequencing data of human ILCs were derived from one experiment but with cells pooled from 10 MISTRG mice that were engrafted with CD34^+^ HSPCs obtained from a total of at least three different human donors. Dimensionality reduction by Uniform Manifold Approximation and Projection (UMAP) revealed eight transcriptionally distinct clusters of 2539 human lung ILCs (Fig. 1A) that expressed characteristic signature genes of ILC1s, ILC2s, and ILC3s (Fig. 1B and Supplementary Table S1).

Cells in cluster 6 transcribed ILC1 signature genes [21, 22], such as the cytokine *IFNG*, the chemokines *CCL4* and *CCL5*, as well as the transcription factor *IKZF3* (Fig. 1B and Supplementary Table S1). Cluster 6 also preferentially expressed the transcription factor *ZNF683* (encoding Hobit), which drives the effector function of ILC1s in mice [23]. Consistent with their ILC1 identity, cells in cluster 6 had the highest gene signature similarity score compared to ILC1s in humans [7] (Fig. 1C and Supplementary Table S2).

**Figure 2:**
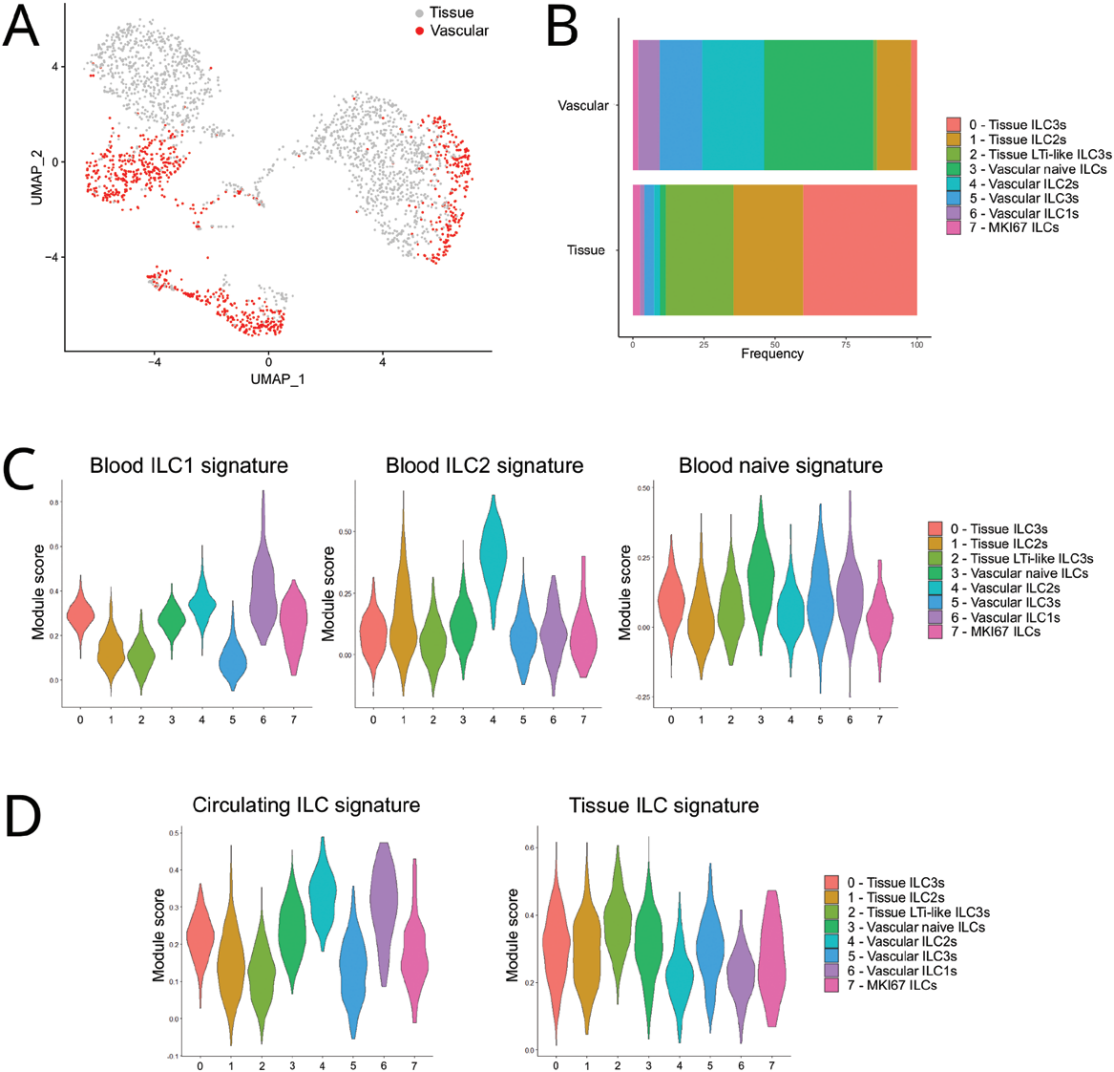
spatial map of human lung ILCs in the vascular and tissue niche. (**A**) Distribution of human ILCs in the lung vasculature and in the lung tissue superimposed on the UMAP from Fig. 1A. (**B**) Relative frequency of the human ILCs clusters in the lung vasculature and in the lung tissue. (**C**) Gene similarity scores of the human lung ILC clusters from HSPC-engrafted MISTRG mice when compared to ILC subsets in human blood. Human blood ILC1, ILC2, and ILC3 signatures are from [7], see Supplementary Table S2. (**D**) Gene signature similarity analysis of human lung ILC clusters from HSPC-engrafted MISTRG mice in comparison to human circulating and tissue ILCs as defined in Ref. [7], see Supplementary Table S2. Data are from one single-cell RNA sequencing experiment with pooled lung cells from 10 MISTRG mice that were transplanted with human CD34^+^ HSPCs from different donors.

Clusters 1 and 4 were identified as ILC2s based on the similarity of their gene signatures to that of human ILC2s [7] (Fig. 1C and Supplementary Table S2). ILC2s in cluster 4 expressed prototypical transcription factors (*GATA3, MAF, BCL11B*), cell surface proteins (*PTGDR2* (encoding CRTH2), *KLRG1*), and other ILC2-associated genes involved in prostaglandin metabolism (*HPGD, HPGDS*) (Fig. 1B and Supplementary Table S1). Like ILC1s (cluster 6), ILC2s (cluster 4) were mostly present in the lung vasculature (Fig. 2A and B) and had greater transcriptional similarity to their counterparts in human blood (Fig. 2C). The other ILC2 cluster (cluster 1) expressed ILC2 genes (such as *KLRG1*) and the transcription factor *RORA* (Fig. 1B and Supplementary Table S1) that is required for the development of ILC2s in mice [24]. The relative frequency of cluster 1 ILC2s was greater in the lung tissue than in the lung vasculature (Fig. 2A and B).

**Figure 3:**
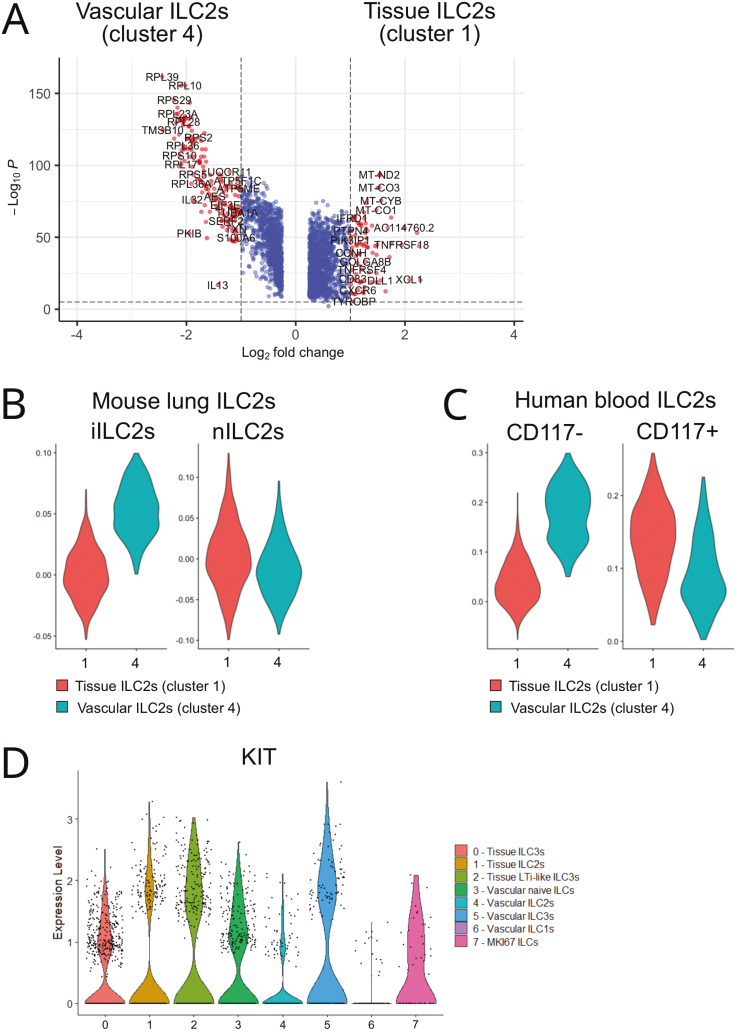
dichotomy of human lung ILC2s in HSPC-engrafted MISTRG mice. (**A**) Volcano plot of genes that are differentially expressed between vascular ILC2s (cluster 4) and tissue ILC2s (cluster 1). (**B** and **C**) Gene module score analysis to determine the similarity of cluster 1 and cluster 4 ILC2s to mouse lung (B) and human blood (C) ILC2s. Gene signatures used for the analysis were obtained from Refs. [25, 50] and Ref.[27], see Supplementary Table S4. (**D**) Violin plot showing *KIT* mRNA expression in human lung ILC clusters from Fig. 1A. Data are from one single-cell RNA sequencing experiment with pooled lung cells from 10 MISTRG mice that were transplanted with human CD34^+^ HSPCs from different donors.

Further analysis revealed that vascular ILC2s in cluster 4 had higher expression of the type 2 cytokine *IL13* and ribosomal genes than tissue ILC2s in cluster 1 (Fig. 3A and Supplementary Table 3). Consistent with their intravascular localization (Fig. 2B) and circulating gene signature (Fig. 2D), cluster 4 ILC2s showed greater transcriptional similarity to mouse iILC2s, known to be mobilized into the systemic circulation upon inflammation [25, 26], than cluster 1 ILC2s (Fig. 3B and Supplementary Table S4). Furthermore, cluster 4 ILC2s were strongly related to canonical c-Kit (CD117)^−^ ILC2s found in human blood (Fig. 3C and Supplementary Table S4). In contrast, the gene signature of lung tissue ILC2s (cluster 1) was more similar to that of CD117^+^ ILC2s (Fig. 3C and Supplementary Table S4) that have been reported to have ILC3-like features [27]. Consistent with this observation, cluster 1 ILC2s had a higher gene similarity score to ILC3s than cluster 4 ILC2s (Fig. 1C and Supplementary Table S2) and expressed significantly more *KIT* mRNA than cluster 4 ILC2s (Fig. 3D and Supplementary Table S3). Overall, these results reveal a dichotomy of human ILC2s in the lung vasculature versus the lung tissue and are consistent with the notion that ILC2s acquire an ILC3-like transcriptional state upon tissue adaptation.

Cells in cluster 2 and 5 transcribed typical ILC3 genes [6, 7, 21, 22, 28, 29], encoding cell surface receptors (*KIT, IL1R1*, *CCR6*), transcription factors (*RBPJ*), and cytokines (*CSF2*, *LTB*) (Figs. 1B and 3D, and Supplementary Table S1). Moreover, the gene signatures of clusters 2 and 5 were similar to the core signature of human ILC3s [7] while having lower similarity scores to ILC1s and ILC2s (Fig. 1C and Supplementary Table S2). ILC3s in cluster 5 were enriched in the lung vasculature but also present in lung tissue, whereas cluster 2 ILC3s were mostly present within the lung tissue (Fig. 2A and B). ILC3s in cluster 5 preferentially expressed *ZBTB16*, encoding the transcription factor PLZF (Supplementary Tables S1 and S5). In mice, ZBTB16 marks local ILC precursors that differentiate into pulmonary ILC3s [30]). Tissue-resident lung ILC3s (cluster 2) had features of lymphoid tissue inducer (LTi)-like ILC3s with preferential expression of MHC class II genes, cytokines of the TNF superfamily (*LTB*, *TNFSF11*, *TNFSF13B*), and surface receptors such as *TNFRSF4* (encoding OX40) and *TNFRSF18* (encoding GITR) (Fig. 1B, Supplementary Fig. S2A, and Supplementary Tables S1 and S5). Cells in cluster 0 were enriched in the lung tissue (Fig. 2A and B), expressed ILC3-related genes (Fig. 1B and Supplementary Table S1), and their gene signature was most similar to ILC3s (Fig. 1C and Supplementary Table S2). Therefore, cluster 0 cells were assigned as tissue ILC3s, although we cannot exclude that they represent contaminating *γδ* T cells with downmodulated CD3 surface protein because they also expressed T cell receptor transcripts, especially *TRDC* (Supplementary Table S1). Cluster 0 tissue ILC3s showed preferential expression of ribosomal genes (Supplementary Figs. S2B and C, Supplementary Table S5).

Cells belonging to cluster 3 were mostly within the lung vasculature (Fig. 2A and B) and did not show any strong similarity with the core signatures of ILC1s, ILC2s, and ILC3s (Fig. 1C and Supplementary Table S2). Cluster 3 ILCs preferentially expressed *TCF7*, *CD7*, and *ILR1* (Fig. 1B and Supplemental Table S1), genes that are characteristic for CD117^+^ ILC precursors [29, 31]. Furthermore, ILCs in cluster 3 had a relatively high similarity score to naïve ILCs in human blood (Fig. 2C). Cells in cluster 3 were, therefore, identified as naïve ILCs. Finally, cluster 7 ILCs were characterized by the expression of proliferation-associated genes, such as *MKI67*, *TOP2A*, and *PCNA* (Fig. 1B and Supplementary Table S1), and were therefore assigned as proliferative ILCs.

ILC2s (cluster 4), ILC1s (cluster 6), and naïve ILCs (cluster 3) possessed gene signatures that were most similar to those of circulating ILCs in humans (Fig. 2D), consistent with their enrichment in the lung vasculature (Fig. 2B). In contrast, the gene signatures of LTi-like ILC3s (cluster 2) and ILC2s (cluster 1) were more similar to those of ILCs in human tissues (Fig. 2D). We conclude that transcriptionally diverse human ILC subsets reside in the vascular and tissue niche of the lung in HSPC-engrafted MISTRG mice.

### Diversity of human lung NK cells in the vascular and tissue compartment

Another member of the ILC family are NK cells that are characterized by their cytotoxic function. For a comparative analysis with the other ILC subsets, we purified human NK cells, defined as CD45^+^Lin^−^CD3^−^TCRαβ^−^CD127^−^CD94^+^ cells, from HSPC-engrafted MISTRG mice to determine their gene signatures in the vascular and tissue compartment of the lung by single-cell RNA sequencing. UMAP analysis revealed six distinct clusters of 3222 human NK cells (Supplementary Fig. S3A and Supplementary Table S6). Gene module score analysis demonstrated that clusters 0, 1, and 3 were most similar to CD56^dim^CD16^+^ NK cells, the predominant NK cell subset in the human lung [32], in terms of their gene signatures (Supplementary Fig. S3B and Supplementary Table S7). In contrast, cells in cluster 4 corresponded to CD56^bright^CD16^-^ NK cells (Supplementary Fig. S3B and Supplementary Table S7). Cluster 2 contained cells that showed intermediate similarity to both CD56^bright^CD16^−^ and CD56^dim^CD16^+^ NK cells (Supplementary Fig. S3B and Supplementary Table S7). Cluster 2 may, therefore, represent a transitional population between the two main NK cell subsets. Finally, there was a cluster of proliferative NK cells (cluster 5) characterized by the expression of *MKI67* and other genes associated with proliferation (Supplementary Fig. S3C and Supplementary Table S6). Proliferative NK cells (cluster 5) were present in both the lung vasculature and lung tissue (Supplementary Fig. S3D and E).

Among the CD56^dim^CD16^+^ NK cell clusters, cluster 0 and 1 preferentially expressed genes encoding cytotoxic molecules, such as perforin, granzymes, and granulysin (Supplementary Fig. S3C and Supplementary Table S6). Furthermore, they transcribed the transcription factor ZEB2 that is associated with the maturation and effector function of CD56^dim^ NK cells [33, 34]. Cluster 1 NK cells were distinguished from cluster 0 NK cells by expressing transcripts encoding MHC class II (Supplementary Table S6). Like Clusters 0 and 1, NK cells in cluster 3 expressed *FCGR3A* mRNA encoding CD16 (Supplementary Fig. S3C). A distinguishing feature of CD56^dim^CD16^+^ NK cell in cluster 3 was the preferential expression of *IFNG* as well as the chemokines *CCL3* and *CCL4* (Supplementary Fig. S3C and Supplementary Table S6Supplementary Table 6). NK cells in clusters 0, 1, and 3 were the predominant NK cell subsets in the lung vasculature of HSPC-engrafted MISTRG mice (Supplementary Fig. S3D and E), consistent with the known abundance of CD56^dim^CD16^+^ NK cells in the circulation [32, 35].

In contrast, CD56^bright^CD16^−^ NK cells (cluster 4) and transitional NK cells (cluster 2) were overrepresented in the extravascular tissue compartment of the lung (Supplementary Fig. S3D and E). CD56^bright^CD16^−^ NK cells in cluster 4 expressed a distinct set of effector molecules, with lower expression of cytolytic genes (Supplementary Fig. S3C). Instead, cluster 4 NK cells preferentially transcribed *TNFSF10* (encoding TRAIL), the cytokines *CSF2*, *LTB*, and *AREG*, as well as the chemokines *XCL1* and *XCL2* (Supplementary Fig. S3C and Supplementary Table S6). Consistent with their enrichment within lung tissue, NK cells in clusters 4 and 5 expressed genes associated with tissue residency, such as *CD44*, *CD69*, *RGS1*, as well as members of the FOS and JUN family of transcription factors (Supplementary Table S6). Taken together, these results show that diverse subsets of human NK cells inhabit distinct anatomical niches within the lung of HSPC-engrafted MISTRG mice.

### Gene signatures of vascular and tissue ILCs in the lung

**Figure 4:**
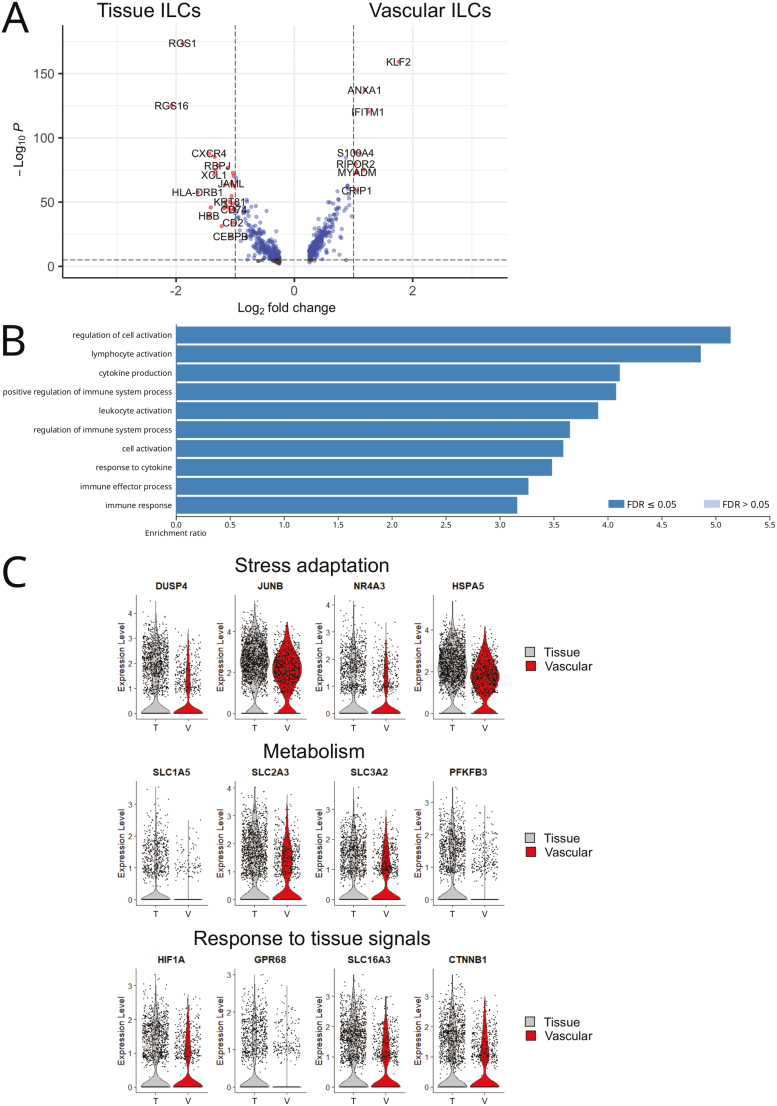
gene signatures of vascular and tissue ILCs in the lung. (**A**) Volcano plot showing differentially expressed genes between vascular and tissue ILCs from the lung of HSPC-engrafted MISTRG mice. (**B**) Gene Ontology over-representation analysis of genes that are upregulated in ILCs located in lung tissue. (**C**) Violin plots of selected genes that are differentially expressed between human ILCs in the lung vasculature and human ILCs in the lung tissue from HSPC-engrafted MISTRG mice. Data are from one single-cell RNA-sequencing experiment with pooled lung cells from 10 MISTRG mice that were transplanted with human CD34^+^ HSPCs from different donors.

To gain insights into how human ILCs adapt to the local microenvironment, we identified genes that were differentially expressed between ILCs in the lung vasculature versus ILCs in the lung tissue. A total of 334 genes were more highly expressed in vascular ILCs, whereas 346 genes had higher expression in tissue ILCs (Supplementary Fig. 4A and Supplementary Table S8). Vascular lung ILCs preferentially transcribed genes that stimulate lymphocyte recirculation [3], such as *SELL* (encoding CD62L), *S1PR1*, *S1PR4*, and the transcription factor *KLF2* (Fig. 4A, Supplementary Fig. S4, and Supplementary Table S8). In addition, other transcripts regulating cell migration (*ITGAL* encoding CD11a, *ICAM2*, *S100A4*, *S100A6*, *S100A10*) were significantly higher in vascular lung ILCs (Fig. 4A and Supplementary Table S8).

Consistent with their anatomical localization, human ILCs within the lung tissue had significantly higher mRNA expression of *CD69*, *ITGAE* (encoding CD103), and *JAML* (Fig. 4A, Supplementary Fig. S4, and Supplementary Table S8), which are prototypical genes associated with tissue residency [3]. Apart from expressing genes promoting lymphocyte retention within tissue, genes involved in lymphocyte adhesion (*ICAM1*, *CD2*, *CD58*, *CD81*), as well as chemotactic receptors (*CXCR4*, *CXCR6*) were also more highly expressed by tissue ILCs in the lung (Fig. 4A, Supplementary Fig. S4 and Supplementary Table S8). These included *GPR183*, a G protein-coupled receptor (GPCR) for cholesterol metabolites that controls ILC3 positioning within tissue [36]. Preferential mRNA expression of these GPCRs was associated with upregulation of genes involved in the regulation of GPCR signaling (*RGS1*, *RGS2*, *RGS9*, *RGS10*, *RGS16*) (Fig. 4A and Supplementary Table S8). Gene Ontology over-representation analysis showed that the gene signature of ILCs in lung tissue was enriched for biological processes related to cell activation and immune effector function (Fig. 4B).

Another category of upregulated transcripts in tissue ILCs was related to adaptation to environmental changes and stress. Specifically, ILCs in lung tissue preferentially expressed immediate early genes (Fig. 4C and Supplementary Table S8) that are induced rapidly without a need for new protein synthesis. These included transcription factors (*JUN*, *JUNB*, *NR4A3*), dual specificity phosphatases (*DUSP2*, *DUSP4*), and heat shock proteins (*HSP90AB1*, *HSPA1A*, *HSPA1B*, *HSPA5*, *HSPD1*, *HSPE1*). Immediate early genes are known to be induced by C/EBP and ATF/CREB transcription factors. Accordingly, *CEBPB* and *CREM* mRNA were significantly higher in ILCs located in the lung tissue (Fig. 4A and Supplementary Table S8). Genes within the NFκB signaling pathway (*MAP3K8*, *NFKBIA*, *REL*) were also induced in tissue ILCs. In addition, genes required for nutrient uptake, specifically encoding glucose (*SLC2A3*) and amino acid transporters (*SLC1A4*, *SLC1A5*, *SLC3A2*, *SLC6A6*, *SLC7A5*), were more highly expressed by tissue ILCs (Fig. 4C and Supplementary Table 8). *SLC3A2* and *SLC7A5* encode CD98 that has been recently shown to promote the proliferation of mouse and human ILC2s [37, 38]. Among metabolic genes, *PFKFB3*, an enzyme involved in glycolysis, was more highly expressed by ILCs in lung tissue (Fig. 4C).

Furthermore, lung tissue ILCs upregulated genes encoding receptors that allow sensing of tissue-derived signals, such as hypoxia (*HIF1A*) and extracellular acidosis (*GPR65*, *GPR68, SLC16A3*), as well as Notch (*RBJP*) and WNT (*CTNNB1*) signals (Fig. 4A and C, and Supplementary Table S8). Recent studies demonstrated that HIF-1α regulates the metabolism, plasticity, and effector function of mouse ILC3s [39–41]. Tissue ILCs in the lung also actively transcribed cytokine receptors (*TNFRSF1B* (encoding TNFR2), *TNFRSF4* (encoding OX40), *TNFRSF18* (encoding GITR), *IL2RB*, *IL4R*, *IL23R*) and transcription factors downstream of cytokine signaling (*STAT1*, *STAT3*, *STAT4*) (Supplementary Fig. S4 and Supplementary Table S8).

Other genes upregulated by tissue ILCs were cytokines of the TNF superfamily, such as *TNFSF10* (encoding TRAIL), *TNFSF13B* (encoding BAFF), *TNFSF14* (encoding LIGHT), and chemokines (*XCL1*, *XCL2*) (Fig. 4A, Supplementary Fig. S4, and Supplementary Table S8) that potentially mediate the interaction of ILCs with other immune cells present in lung tissue, for example, B lymphocytes [42] and dendritic cells. Consistent with this notion, tissue ILCs in the lung also had higher expression of MHC class II genes (e.g. *HLA-DRA*, *HLA-DRB1*) (Fig. 4A and Supplementary Table S8), which could support their interaction with CD4 T cells in analogy to what has been reported for mouse ILC3s [43]. Combined, these findings show that tissue-resident human ILCs acquire a gene expression program that likely promotes their adaptation to the extravascular space of the lung.

### Subset-specific and shared signatures of tissue ILC2s and ILC3s in the lung

**Figure 5:**
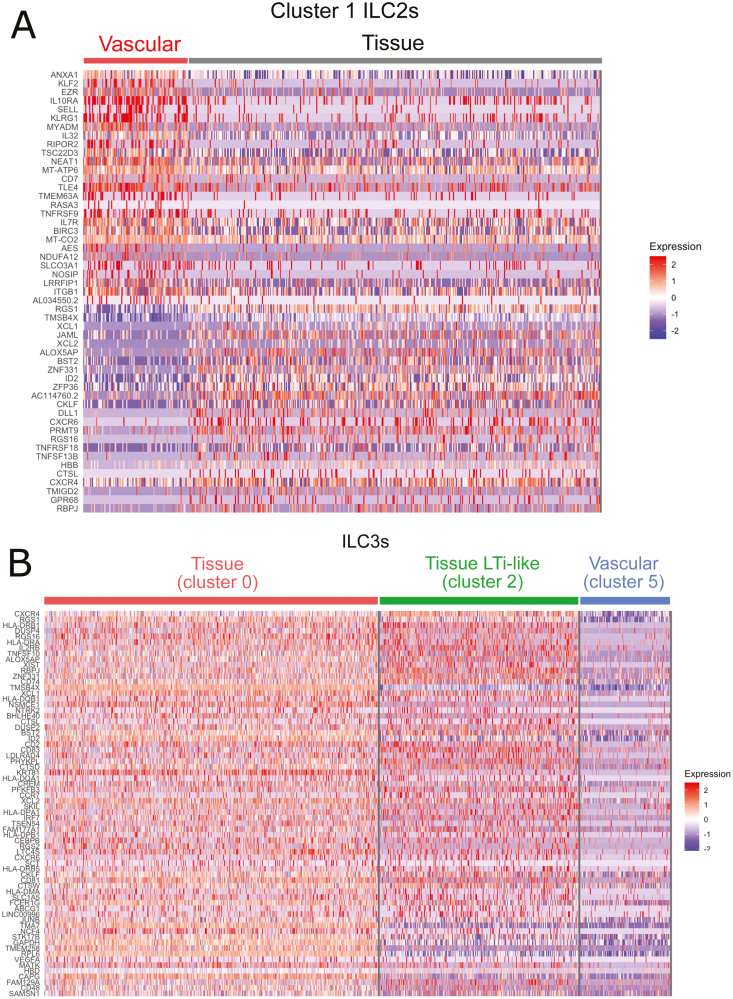
subset-specific transcriptional profiles of tissue-resident lung ILC2s and ILC3s. (**A**) Heatmap showing genes differentially expressed between vascular and tissue ILC2s within cluster 1. (**B**) Heatmap of genes that are more highly expressed by tissue ILC3s (cluster 0 and cluster 2) than by vascular ILC3s (cluster 5). Data are from one single-cell RNA-sequencing experiment with pooled lung cells from 10 MISTRG mice that were transplanted with human CD34^+^ HSPCs from different donors.

Cluster 1 ILC2s were predominantly within lung tissue, but also contained cells in the lung vasculature (Fig. 2A and B). This enabled us to compare the transcriptional profiles of vascular versus tissue ILC2s within the same cluster. We found that vascular cluster 1 ILC2s preferentially transcribed genes associated with circulating cells (*KLF2*, *SELL*, *ITGB1*) and other genes, such as *CD7*, *KLRG1*, *IL7R*, and *IL10RA* (Fig. 5A). In contrast, their counterparts in the lung tissue showed higher expression of genes associated with tissue residency (*JAML*, *CXCR4*, *CXCR6*, *RGS1*, *RGS16*), as well as chemokines (*XCL1*, *XCL2*, *CKLF*), TNF receptor superfamily members (*TNFRSF13B*, *TNFRSF18*), and the transcription factor *RBPJ* (Fig. 5A).

To interrogate ILC3-specific signatures, we identified genes that were more highly expressed by vascular ILC3s (cluster 5) than by ILC3s in the lung tissue (cluster 0 and 2). Similar to vascular ILC2s (Fig. 5A), ILC3s in the lung vasculature (cluster 5) expressed more *KLF2*, *CD7*, and *CD55* mRNA than cluster 0 and cluster 2 ILC3s located in the lung tissue (Supplementary Fig. S5). On the other hand, tissue ILC3s (cluster 0 and 2) more highly expressed MHC class II genes and genes encoding other surface proteins (*CD2*, *CD81*, *CD83*, *CCR7*, *IL2RB*) (Fig. 5B). Several genes (e.g. *CXCR4*, *CXCR6*, *RGS1*, *RGS16*, *XCL1*, *XCL2*, *CKLF*, *RBPJ, ID2*) were shared between ILC2s and ILC3s residing in the lung tissue (Fig. 5A and B).

### Transcriptional profiles of human NK cells in the lung vasculature and lung tissue

We performed a similar analysis of NK cells, which identified 176 and 268 genes that were significantly more highly expressed by vascular and tissue NK cells, respectively (Supplementary Table S9). NK cells in the lung vasculature had higher expression of genes involved in recirculation (*KLF2*, *S1PR5*) and cell adhesion (*ITGB2*, *ADGRG1*, *CX3CR1*) (Supplementary Fig. S6 and Supplementary Table 9). Consistent with enrichment of CD56^dim^CD16^+^ NK cells in the vasculature, *FCGR3A* (encoding CD16) as well as cytotoxic genes, chemokines (*CCL3*, *CCL4*, *CCL5*), and transcription factors promoting terminal NK cell differentiation (*ZEB2*, *PRDM1*) showed higher expression in vascular than in tissue NK cells within the lung (Supplementary Fig. S6 and Supplementary Table S9).

As expected, NK cells residing in the lung tissue, had higher expression of genes associated with tissue residency (*CD44*, *CD69*) (Supplementary Fig. S6 and Supplementary Table S9). Similar to lung-resident ILCs, NK cells in lung tissue preferentially expressed immediate early genes, such as transcription factors (*FOS*, *FOSB*, *JUN*, *JUND*, *CEBPB*) and dual specificity phosphatases (*DUSP4*) (Supplementary Fig. S6 and Supplementary Table 9). Likewise, tissue NK cells transcribed receptors to sense hypoxia (*HIF1A*) and changes in extracellular pH (*GPR65*). In addition, tissue NK cells expressed genes transducing Notch and WNT signals (*RBJP*, *CTNNB1*). Whereas NK cells in the lung vasculature upregulated genes encoding MHC class I proteins, NK cells in lung tissue had higher expression of MHC class II genes (Supplementary Table 9). Unlike cytolytic NK cells in the lung vasculature, tissue NK cells expressed cytokines and chemokines (*CSF2*, *MIF*, *CKLF*, *XCL1*, *XCL2*) acting on mononuclear phagocytes (Supplementary Fig. S6 and Supplementary Table S9). Finally, tissue-resident NK cells in the lung preferentially expressed mRNA for the surface proteins *CD2*, *CD81*, and *TNFRSF18* (encoding GITR) (Supplementary Fig. S6 and Supplementary Table S9).

### Shared molecular features of human ILCs and NK cells located in lung tissue

To define the core signature of lung-resident ILCs and NK cells, we focused on upregulated genes that were common to both ILCs and NK cells located within the lung tissue. A total of 53 transcripts were significantly higher in vascular than in tissue ILCs and NK cells (Supplementary Fig. S7). Consistent with their localization, ILCs and NK cells in the lung vasculature preferentially transcribed genes involved in lymphocyte recirculation, migration, and adhesion, such as members of the Krüpple-like transcription factor family (*KLF2*, *KLF3, KLF6*), S100A proteins *S100A4*, *S100A10*), and *ITGAL* (Supplementary Fig. S7).

There were 65 genes that were commonly upregulated by both tissue ILCs and NK cells in the lung (Supplementary Fig. S8). These included genes required for lymphocyte retention within tissue (*CD69*) as well as genes regulating GPCR signaling (*RGS1*, *RGS2*, *RGS9*, *RGS10*, *RGS16*) downstream of migratory receptors (Supplementary Fig. S8). Other upregulated genes were involved in the adaptation of cells to stress, specifically immediate early genes (*JUNB*, *DUSP4*) and transcription factors regulating their expression (*CEBPB*, *NFKBIA*, *REL*), as well as heat shock proteins (*HSP90AB1*) (Supplementary Fig. S8). Another prominent group of genes was related to the sensing of tissue-derived signals, such as hypoxia (*HIF1A*), tissue acidosis (*GPR65*), cytokines (*IL2RB*, *TNFRSF4*, *TNFRSF18*), as well as Notch and WNT signals (*RBJP*, *CTNNB1*) (Supplementary Fig. S8). Finally, genes mediating the interaction with lymphocytes and myeloid cells were also more highly expressed by human ILCs and NK cells within the lung tissue. These included cytokines regulating the function of B lymphocytes (*TNFSF14*), chemokines to recruit myeloid cells (*XCL1*, *XCL2*, *CKLF*), and MHC class II genes (*HLA-DQA1*, *HLA-DRA*) promoting the interaction with CD4 T lymphocytes (Supplementary Fig. S8). In summary, our results indicate that human ILCs and NK cells react to niche-specific cues when they migrate from the local vasculature into the lung tissue.

### Surface protein expression in tissue-adapted human lung ILCs and NK cells

**Figure 6:**
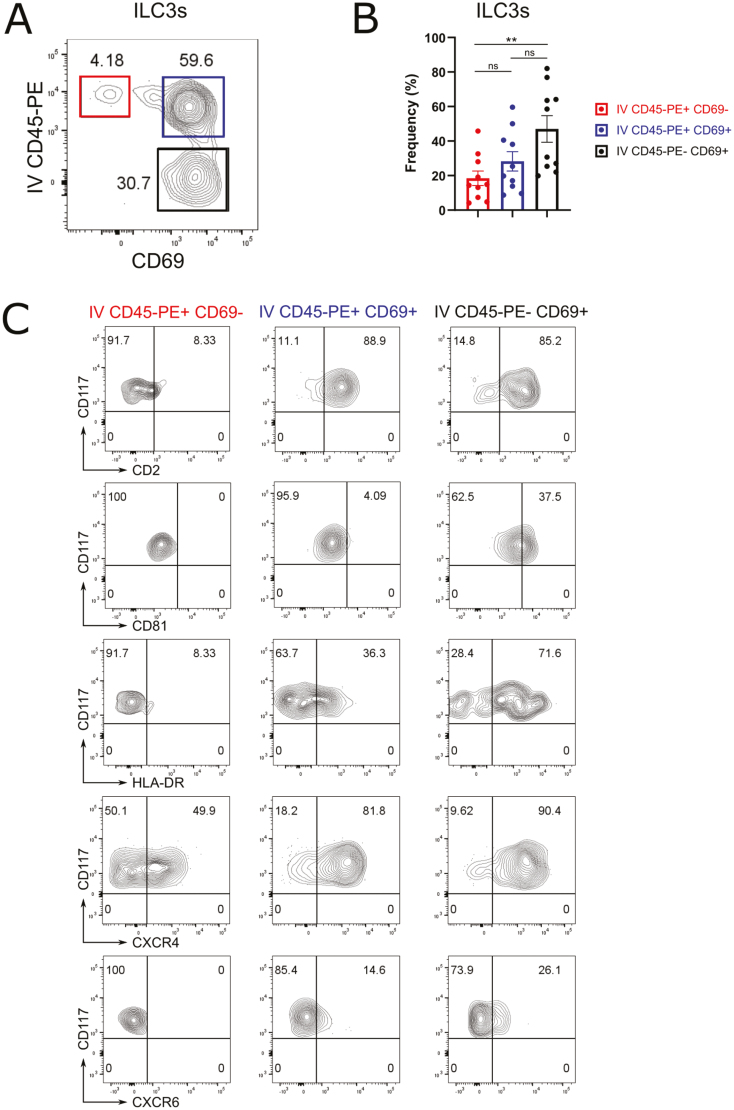
surface protein expression in tissue-adapted human lung ILC3s. (**A**) Flow cytometry of lung ILC3s from HSPC-engrafted MISTRG mice. Contour plots show IV CD45-PE labeling and CD69 surface expression. ILC3s were gated as CD45^+^Lin^−^CD3^−^TCRαβ^−^CD127^+^CD94^−^CD117^+^CRTH2^−^ cells as in Supplementary Fig. S1A. (**B**) Frequency of IV CD45-PE^+^CD69^−^, IV CD45-PE^+^CD69^+^, and IV CD45-PE^-^CD69^+^ cells among lung ILC3s. Error bars indicate SEM. n.s., not significant; **P < 0.01 by one-way ANOVA with post hoc testing. (**C**) Surface expression of CD2, CD81, HLA-DR, CXCR4, and CXCR6 on human lung ILC3s according to IV CD45-PE^+^CD69^−^, IV CD45-PE^+^CD69^+^, and IV CD45-PE^-^CD69^+^ subsets. Data are representative of 4–5 independent experiments (*n* = 8–10 mice). The CXCR4 data are representative of two independent experiments (*n* = 3 mice).

Next, we performed flow cytometry to confirm selected genes encoding surface proteins in tissue-resident lung ILCs. For these experiments, we focused on ILC3s since they were the most prevalent ILC subset in the lung (Supplementary Fig. S1A and B). Combining intravascular cell labeling with surface staining for CD69 allowed us to distinguish three ILC3 populations in the lung of HSPC-engrafted MISTRG mice: As expected, extravascular lung ILC3s that were not labeled by the IV CD45-PE antibody uniformly expressed CD69 on their cell surface (Fig. 6A and B), identifying them as tissue-resident IV CD45-PE^−^CD69^+^ ILC3s. Interestingly, intravascular lung ILC3s that were IV CD45-PE^+^ contained not only CD69^−^ but also CD69^+^ ILC3s (Fig. 6A and B), consistent with the idea that CD69 upregulation occurs already in the lung vasculature. We observed a similar pattern for NK cells, but they were mostly IV CD45-PE^+^CD69^+^ with fewer IV CD45-PE^−^CD69^+^ cells (Supplementary Fig. S9A and B).

We found that CD2 and CXCR4 surface proteins were upregulated on both IV CD45-PE^+^CD69^+^ and IV CD45-PE^-^CD69^+^ ILC3s (Fig. 6C). On the other hand, the tetraspanin CD81 as well as HLA-DR, the chemokine receptor CXCR6, and the amino acid transporter CD98 were predominantly expressed by IV CD45-PE^−^CD69^+^ ILC3s (Fig. 6C and Supplementary Fig. S10). Overall, these results are consistent with differential mRNA expression of *CD2*, *CD81*, *HLA-DRB1*, *CXCR4*, *CXCR6*, and *SLC3A2/SLC7A5* (encoding CD98) in lung tissue ILC3s (Fig. 5B and Supplementary Table S8). In addition, IV CD45-PE^+^CD69^+^ and to a lesser extent IV CD45-PE^-^CD69^+^ ILC3s expressed CD56 on their cell surface (Supplementary Fig. S10), a marker for CD117^+^ ILCs committed to the ILC3 (or NK cell) fate [29, 44]. In contrast, the “naïve” ILC marker CD45RA [31] was mostly expressed by IV CD45-PE^+^CD69^−^ ILC3s and downmodulated on IV CD45-PE^-^CD69^+^ ILC3s (Supplementary Fig. S10).

Similar to ILC3s, IV CD45-PE^-^CD69^+^ tissue NK cells had higher CD2, CD81, and CD98 surface expression than vascular (IV CD45-PE^+^) lung NK cells (Supplementary Fig. S9C). IV CD45-PE^-^CD69^+^ tissue NK cells also expressed more GITR surface protein (encoded by *TNFRSF18*) (Supplementary Fig. S9C), in agreement with higher mRNA expression of *CD2* and *TNFRSF18* in NK cells that were located in lung tissue (Supplementary Fig. S6 and Supplemental Table S9). Consistent with the single-cell RNA-sequencing data, the IV CD45-PE^−^CD69^+^ NK cells in lung tissue were mostly CD16^−^ (Supplementary Fig. S9C), equivalent to the CD56^bright^ subset. Finally, IV CD45-PE^-^CD69^+^CD16^−^ NK cells in lung tissue mostly lacked CD45RA surface protein (Supplementary Fig. S9C). Taken together, these data indicate that human ILC3s and NK cells share conserved features when adapting to the extravascular tissue microenvironment in the lung.

## Discussion

Human ILCs acquire tissue-specific phenotypes in response to cues from the local microenvironment [6, 7, 10, 32]. Furthermore, ILC function is regulated by their anatomical distribution and localization within specific tissue niches [9]. In this study, we defined the molecular programs underlying the adaptation of human lung ILCs to their microenvironment, using a humanized mouse model. We employed single-cell RNA sequencing combined with intravascular cell labeling to resolve the cellular and anatomical heterogeneity of human ILCs and NK cells in the lung. We demonstrate that human ILCs occupy distinct niches in the lung, which imprint niche-specific transcriptional programs. Furthermore, a comparative analysis with human NK cells uncovered molecular pathways that are shared between human ILCs and NK cells residing within the lung tissue, independent of subset-specific transcriptional profiles. Overall, our study contributes to understanding how human ILCs undergo tissue adaptation in the lung.

All main populations of human ILCs and NK cells were present in the lung of HSPC-engrafted MISTRG mice at a similar frequency than in the human lung [7, 14]. Heterogenous ILC and NK cell populations resided in the lung vasculature and the lung tissue. The transcriptional heterogeneity and distinct gene signatures of vascular versus tissue NK cells largely corresponded to the anatomical demarcation of the two main NK subsets. Specifically, CD56^dim^ cytotoxic NK cells were largely confined to the lung vasculature, whereas CD56^bright^ NK cells were enriched in lung tissue, expressing a different profile of effector genes. Compared with NK cells, the main helper-like ILC subsets were present in both the vascular and tissue space of the lung in HSPC-engrafted MISTRG mice.

Our findings indicate that the intravascular and extravascular space constitute distinct ILC niches in the lung, which imprint niche-specific transcriptional programs. This imprinting may represent a gradual process during and after extravasation of ILCs into the lung tissue. We cannot formally distinguish whether the intravascular ILCs in HSPC-engrafted MISTRG mice are marginated in the lung vasculature or are recirculating. However, human ILC3s in the lung vasculature had higher CD69 surface expression than their counterparts in the blood and CD69 distinguished two ILC3 populations in the lung vasculature. This is consistent with the notion that CD69^+^ ILC3s spend a considerable amount of time in the lung vasculature and that intravascular CD69^+^ ILC3s within the lung are different from their counterparts in the systemic circulation. Furthermore, it suggests that ILC3s already start to acquire lung-specific traits when they marginate in the local vasculature.

Our results demonstrate that ILC2s in the lung vasculature corresponded to canonical ILC2s expressing core genes associated with human blood ILC2s. Furthermore, vascular lung ILC2s had higher mRNA expression of the classical type 2 effector cytokine IL-13. In contrast, extravascular ILC2s in the lung tissue had a distinct transcriptional profile. They showed ILC3-like features and were similar to previously described CD117^+^ ILC2s found in human blood and skin [27]. These findings suggest that, at least in steady-state conditions, the tissue microenvironment in the lung may favor an ILC3-related transcriptional program.

Comparing their single-cell gene signatures revealed specific molecular attributes of human ILCs localized within the lung tissue of HSPC-engrafted MISTRG mice. We identified genes with diverse functions in the establishment of tissue residency and in the adaptation to the tissue environment of the lung. As a likely first step in becoming tissue-resident cells in the lung, human ILCs upregulated genes regulating cell migration. These included genes mediating ILC homing and extravasation into the lung, as well as directing ILC positioning within the lung tissue. Consistent with their tissue-resident phenotype, ILCs within lung tissue also preferentially expressed genes promoting their retention within the lung, such as *CD69*. Furthermore, our analysis suggests that once ILCs are in the tissue space of the lung, they are likely poised to respond to tissue-derived signals and environmental challenges. For example, we found that ILCs within lung tissue are equipped with receptors that sense alterations of lung homeostasis due to hypoxia and tissue acidosis. In addition, tissue ILCs in the lung preferentially expressed receptors and transcription factors that make them responsive to cytokine, Notch, and WNT signals. Another feature of ILCs residing within the extravascular compartment of the lung was the expression of immediate early genes that are transcribed independently of new protein synthesis. This likely enables ILCs within tissue to rapidly react to disturbed lung homeostasis and to adapt to stress conditions within the inflamed lung. ILCs within lung tissue also had a distinctive profile in terms of metabolism. They upregulated genes promoting the uptake of nutrients, such as amino acids and glucose, which likely helps ILCs to adjust to the specific metabolic milieu in the lung tissue. Another feature of tissue-adapted human ILCs in the lung was the expression of genes (MHC class II, cytokines, chemokines) known to be involved in the interaction with other tissue-resident immune cells, such as CD4 T lymphocytes [43] and myeloid cells.

Our work complements a recent study that performed single-cell RNA sequencing of ILCs in the human lung [7]. However, the study by Mazzurana et al. did not distinguish between ILCs in the lung vasculature versus ILCs in the lung tissue. Therefore, the reported ILC signatures in the human lung represent a composite. We used the MISTRG humanized mouse model to take into account the spatial compartmentalization of ILCs into vascular and tissue niches. While the MISTRG model allows studies of human ILCs *in vivo* that would otherwise not be possible, the model has limitations. For example, human ILCs and other human immune cells in MISTRG mice interact with the mouse endothelium, stroma, and epithelium of the lung. As some factors and their receptors are not cross-reactive between humans and mice, cellular interactions between human ILCs and mouse non-hematopoietic cells may be suboptimal. However, the organizational structure of the lung and its cell–cell communication hubs are largely conserved between humans and mice [45]. Furthermore, human ILCs in HSPC-engrafted MISTRG mice were able to migrate into the extravascular compartment of the mouse lung, suggesting that their migration is intact. Finally, human ILC subsets in the MISTRG lung showed a similar composition than in the human lung, with a predominance of ILC3s instead of ILC2s. Despite its limitations, our study provides important information on the biology of human ILCs that is complementary to studies of human lung tissue. In particular, our study sheds light on the likely mechanisms of ILC tissue adaptation in the lung. Furthermore, our results open avenues for future investigation, for example, to validate cell surface receptors regulating the extravasation of human ILCs from the local vasculature and their subsequent positioning within lung tissue. ILCs have been implicated in tissue pathology and the niche-specific molecular profiles in this study could pinpoint molecular targets to modulate ILC function in the context of human lung diseases.

## Methods and materials

### Humanized mice

MISTRG mice, genetically modified to express human cytokines, have been described previously [19, 46]. Briefly, MISTRG mice lack mouse *recombination activating gene 2* (*Rag2*) and *IL-2 receptor gamma* (*Il2rg*) and have human genes knocked-in that encode the proteins macrophage colony-stimulating factor (M-CSF), IL-3/granulocyte-macrophage colony-stimulating factor (GM-CSF), signal-regulatory protein alpha (SIRPα), and thrombopoietin (TPO). For transplantation with human CD34^+^ HSPCs (see below), MISTRG mice heterozygous for the *SIRPA* knock-in and homozygous for all other human genes were used as in our previous studies [17, 18, 20, 47]. MISTRG mice were housed in individually ventilated cages without using prophylactic antibiotics, except for a 3-week period after irradiation and HSPC transplantation (see below). Both male and female MISTRG mice were used for experiments. All animal experiments were carried out according to protocols approved by the Linköping Ethics Committee (#29-15 and 03127-2020). Karolinska Institutet’s guidelines on the care and use of laboratory animals were followed. Material Transfer Agreements with Regeneron Pharmaceuticals and Yale University are required to use MISTRG mice.

### Transplantation of MISTRG mice with human CD34^+^ HSPCs

As a source of human HSPCs, CD34^+^ cells were isolated from umbilical cord blood as described [17]. The collection of human umbilical cord blood was approved by the Ethical Review Board at Karolinska Institutet (#2015/1368-31/4, 2015/2122-32, 2019-00618) and informed consent was obtained after giving verbal and written information according to the Declaration of Helsinki. For the single-cell RNA-sequencing experiments, newborn MISTRG mice were pre-conditioned by irradiation (100 cGy) and transplanted with pooled batches of human CD34^+^ cells by intrahepatic injection as described [18]. MISTRG mice received prophylactic antibiotics (Bactrim) in the drinking water for 3 weeks after irradiation and transplantation. For the flow cytometry experiments, MISTRG mice were transplanted without any pre-conditioning by irradiation.

### Purification of human ILCs and NK cells from MISTRG mice

To distinguish between blood-borne (intravascular) and tissue-resident (extravascular) ILC populations within the lung, we performed intravascular cell labeling. A PE-conjugated anti-human CD45 antibody (clone HI30, Biolegend) was IV injected into MISTRG mice transplanted with human CD34^+^ HSPCs as described [17, 18, 20]. We used saturating amounts of the antibody (2 μg) for our study (Supplementary Fig. S1D). Lungs obtained from HSPC-engrafted MISTRG mice were rinsed with medium to wash out any unbound anti-human CD45-PE antibody before processing the lungs by enzymatic digestion as described [17, 18]. After antibody injection, lung lymphocytes were stained *ex vivo* with ILC-specific antibodies to purify intravascular (IV CD45-PE^+^) and extravascular (IV CD45-PE^-^) human ILCs and NK cells by cell sorting. Before cell sorting, lung ILCs were enriched by negative immunomagnetic selection with a cocktail of biotin-conjugated antibodies (CD3, CD14, CD16, CD19) and Mojosort Streptavidin Nanobeads (Biolegend) using the protocol by Krabbendam et al. [48]). ILCs were then sort-purified as human CD45^+^CD127^+^CD94^−^ cells. NK cells were purified as human CD45^+^CD127^−^CD94^+^ cells in a separate experiment with different MISTRG mice. Other immune cells were excluded by using antibodies against T cell surface markers (CD3, TCRαβ) and the Lin markers CD11c, CD14, CD19, CD123, and FceRI. To obtain enough human ILCs and NK cells, lung cells from several HSPC-engrafted MISTRG mice were pooled for two separate experiments: (1) lung cells from 10 mice for the ILC single-cell RNA-sequencing experiment; (2) lung cells from 9 mice for the separate NK cell single-cell RNA-sequencing experiment. MISTRG mice engrafted with different batches of cord blood CD34^+^ cells (from a total of at least three donors per single-cell RNA-sequencing experiment) were used to account for human HSPC donor variability.

### Single-cell RNA sequencing of human ILCs and NK cells

RNA extraction and library preparation from purified ILCs and NK cells were performed according to Drop-Seq protocols from 10× Genomics with the Single Cell 3’ Library v2 kit. Sequencing of libraries, mapping to the human genome, and analysis with Seurat 4.2.1 was performed essentially as described [18]. Briefly, after selecting genes expressed in ≥3 cells and cells expressing ≥200 genes, the vascular (IV CD45-PE^+^) and tissue (IV CD45-PE^−^) ILC samples were merged into a single Seurat object as were the vascular (IV CD45-PE^+^) and tissue (IV CD45-PE^−^) NK cell samples. Then, cells with >100 genes, <5000 (ILC dataset) or <7000 genes (NK cell dataset), and <5% (NK cell dataset) or <10% (ILC dataset) mitochondrial genes were selected. After log normalization to 10,000 counts, 2000 highly variable genes were chosen to generate PCA and neighborhood graphs. Initial clustering was performed with Louvain = 0.5 (for ILCs) and Louvain = 0.45 (for NK cells). Next, contaminating clusters of red blood cells and monocytes/dendritic cells (ILC dataset) or macrophages (NK cell dataset) were removed. Then, Louvain reclustering with resolution 0.6 (ILCs) or 0.5 (NK cells) was performed to create the UMAPs shown in Fig. 1A and Supplementary Fig. S3A. This resulted in 2539 ILCs (869 vascular ILCs, 1670 tissue ILCs) and in 3222 NK cells (2508 vascular NK cells, 714 tissue NK cells). Marker genes for each ILC or NK cell cluster were computationally identified using min.pct = 0.25 and logfc.threshold = 0.25. Differentially expressed genes between vascular and tissue ILCs or NK cells were determined using MAST [49]. Genes with adjusted *P* values <0.05 were considered statistically significant. Dot plots, Violin plots, Volcano plots, and heatmaps were generated with built-in functions in Seurat. Gene module score analysis was performed in Seurat with the GeneModuleScore function, using published single-cell and bulk RNA-sequencing datasets: (i) ILCs from human lung, blood, and other tissues [7] for Fig. 1C, 2C, and 2D; (ii) Human CD56^dim^CD16^+^ and CD56^bright^CD16^−^ NK cells [32] for Supplementary Fig. S3B; (iii) Mouse lung iILC2s and activated nILC2s [25, 50], for Fig. 3B; (iv) Human CD117^−^ and CD117^+^ blood ILC2s [27] for Fig. 3C. The individual gene signatures used for Gene module score analysis are shown in Supplementary Tables S2, S4, and S7. For Gene Ontology over-representation analysis (Biological Process), WebGestalt (https://www.webgestalt.org/) was used with default parameters.

### Flow cytometry of human ILCs and NK cells

Lung cells from HSPC-engrafted MISTRG mice were isolated as described above. Cell surface staining with fluorescent antibodies was performed as previously described [17, 18]. The following antibodies were used for flow cytometry: CD127 (clone A019D5, Biolegend), CRTH2 (clone BM16, Biolegend), CD94 (clone DX22, Biolegend), CXCR4 (clone 12G5, Biolegend), CXCR6 (clone K041E5, Biolegend), GITR (clone 108-17, Biolegend), CD16 (clone 3G8, Biolegend), CD69 (clone FN50, Biolegend), CD117 (104D2D1, Beckman Coulter), HLA-DR (clone G46-6, BD Biosciences), CD2 (clone RPA-2.10, BD Biosciences), CD56 (clone NCAM16.2, BD Biosciences), CD81 (clone JS-81, BD Biosciences), CD94 (clone HP-3D9, BD Biosciences), CD98 (clone UM7F8, BD Biosciences), CD103 (clone Ber-ACT8, BD Biosciences), and CD45RA (clone HI100, BD Biosciences). In all experiments, ILCs were defined as human CD45^+^Lin^-^CD3^−^TCRαβ^−^CD127^+^CD94^−^ cells. Lineage markers included CD14 (clone M5E2, Biolegend), CD19 (clone HIB19, Biolegend), CD11c (clone Bu15, Biolegend), CD123 (clone 6H6, Biolegend), FcεRIα (clone AER-37 (CRA-1), Biolegend), CD34 (clone 581, BD Biosciences), TCRαβ (clone IP26) and CD3 (clone SK7).

### Statistical analysis

Data are shown in Fig. 6B and Supplementary Fig. S1B, S1F, and S9B with mean and standard error of the mean (SEM). The number of replicates, independent experiments, and statistical tests used are described in the figure legends. For statistical analysis GraphPad Prism 9 (https://www.graphpad.com/scientific-software/prism/) was used with α = 0.05 considered statistically significant. One-way ANOVA with post hoc testing using Tukey’s multiple comparison test was applied.

## Supplementary Material

kyad007_suppl_Supplementary_Figures

kyad007_suppl_Supplementary_Table_S1

kyad007_suppl_Supplementary_Table_S2

kyad007_suppl_Supplementary_Table_S3

kyad007_suppl_Supplementary_Table_S4

kyad007_suppl_Supplementary_Table_S5

kyad007_suppl_Supplementary_Table_S6

kyad007_suppl_Supplementary_Table_S7

kyad007_suppl_Supplementary_Table_S8

kyad007_suppl_Supplementary_Table_S9

## Data Availability

The single-cell RNA-sequencing data (read counts) underlying this article are available in the Gene Expression Omnibus (GEO) repository at https://www.ncbi.nlm.nih.gov/geo/ under the accession number GSE217706. The raw sequencing data cannot be shared publicly in order to protect the identity of the human cord blood donors as required by Swedish legislation and the European General Data Protection Regulation (GDPR).

## Abbreviations:

GPCR: G protein-coupled receptor
HSPCs: hematopoietic stem and progenitor cells
ILCs: innate lymphoid cells
IV: intravenous
Lin: lineage
LTi-like: lymphoid tissue inducer-like
NK: natural killer
PE: phycoerythrin
UMAP: Uniform Manifold Approximation and Projection
