# Supplementary material for: Single-cell RNA sequencing of human lung innate lymphoid cells in the vascular and tissue niche reveals molecular features of tissue adaptation
Source: Discov Immunol. 2023 Jun 24;2(1):kyad007. doi: 10.1093/discim/kyad007 (PMC11034571; doi:10.1093/discim/kyad007)
Supplement: kyad007_suppl_Supplementary_Figures [file kyad007_suppl_Supplementary_Figures.pdf]

Figure S1

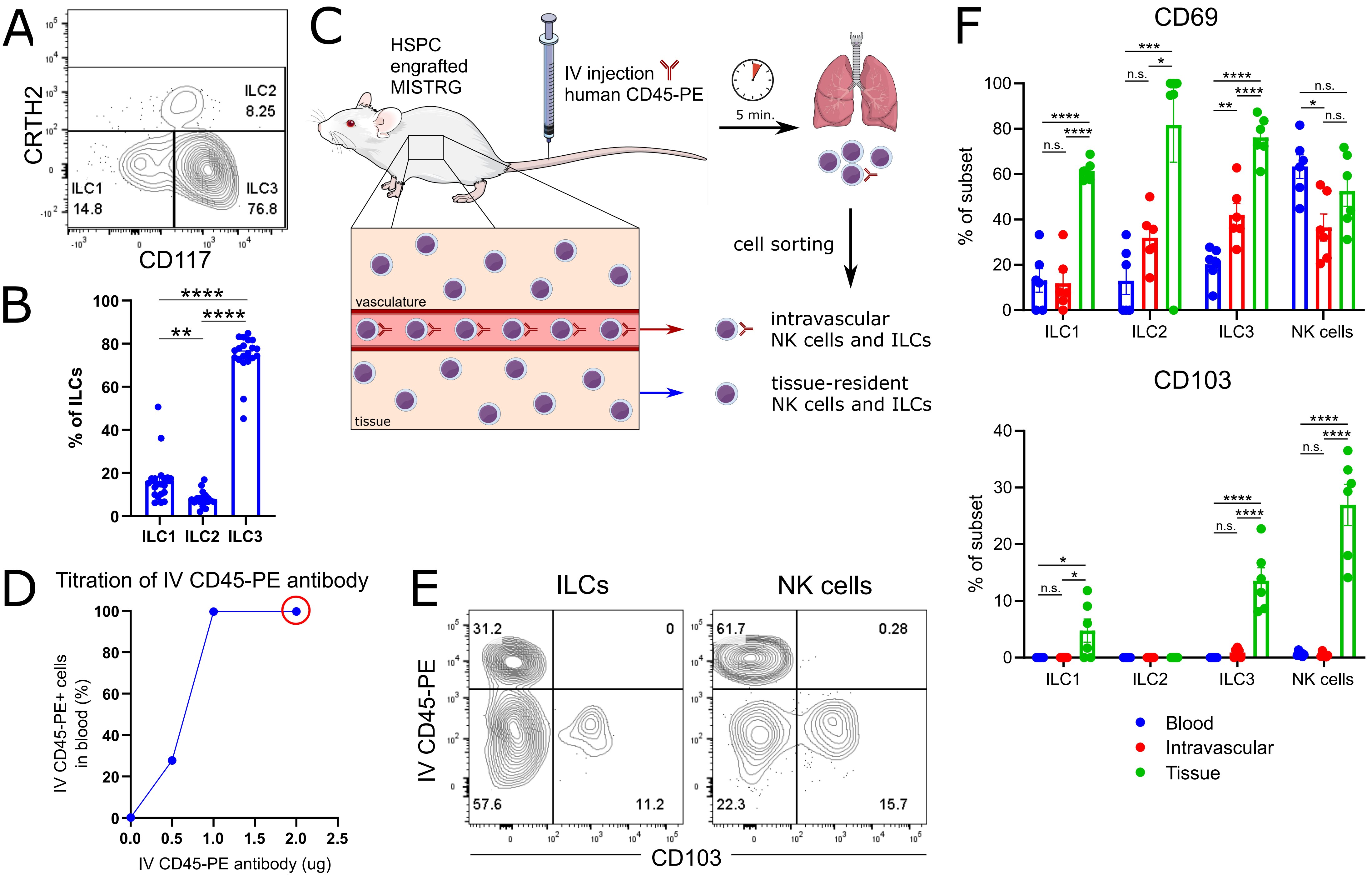

A
CRTH2
CD117
ILC2
8.25
ILC1
14.8
ILC3
76.8
B
% of ILCs
ILC1 ILC2 ILC3
C
HSPC engrafted MISTRG
IV injection human CD45-PE
5 min.
cell sorting
vasculature
tissue
intravascular NK cells and ILCs
tissue-resident NK cells and ILCs
D
Titration of IV CD45-PE antibody
IV CD45-PE+ cells in blood (%)
IV CD45-PE antibody (ug)
E
ILCs
NK cells
IV CD45-PE
CD103
31.2
0
57.6
11.2
61.7
0.28
22.3
15.7
F
CD69
CD103
% of subset
ILC1 ILC2 ILC3 NK cells
Blood
Intravascular
Tissue

Fig. S1. Visualizing vascular and tissue ILCs in the lung of HSPC-engrafted MISTRG mice. (**A-B**) Flow cytometry and frequency of human $CD45^{+}Lin^{-}CD3^{-}TCRab^{-}CD127^{+}CD94^{-}$ ILCs in the lung of HSPC-engrafted MISTRG mice (n=20). Lin markers included CD11c, CD14, CD19, CD123, and FceRI. ILC subsets were gated as shown in (A). (**C**) Overview of intravascular cell labeling to distinguish intravascular from extravascular (tissue-resident) human ILCs and NK cells in the lung. Lungs were harvested from HSPC-engrafted MISTRG mice 5 minutes (min.) after IV injection of anti-human CD45-PE antibody. The cartoon was created with Mind the Graph. (**D**) Binding of IV-injected CD45-PE antibody to circulating human hematopoietic cells in HSPC-engrafted MISTRG mice. The amount of IV CD45-PE antibody used for subsequent experiments (2mg) is indicated by the red circle. (**E**) Flow cytometry of human ILCs ($CD127^{+}CD94^{-}$) and NK cells ($CD127^{-}CD94^{+}$) from the lung of HSPC-engrafted MISTRG mice after intravascular labeling with IV CD45-PE antibody. The frequencies of CD103-expressing cells are shown. (**F**) Frequency of $CD69^{+}$ and $CD103^{+}$ ILC subsets and NK cells from the blood, intravascular compartment of the lung, and tissue compartment of the lung of HSPC-engrafted MISTRG mice (n=6). Error bars indicate SEM. n.s., not significant; *, P<0.05; **, P<0.01; ***, P<0.001; ****, P<0.0001 by one-way ANOVA with post hoc testing. Data are from 2 (E and F) or 5 (A and B) independent experiments.

Figure S2

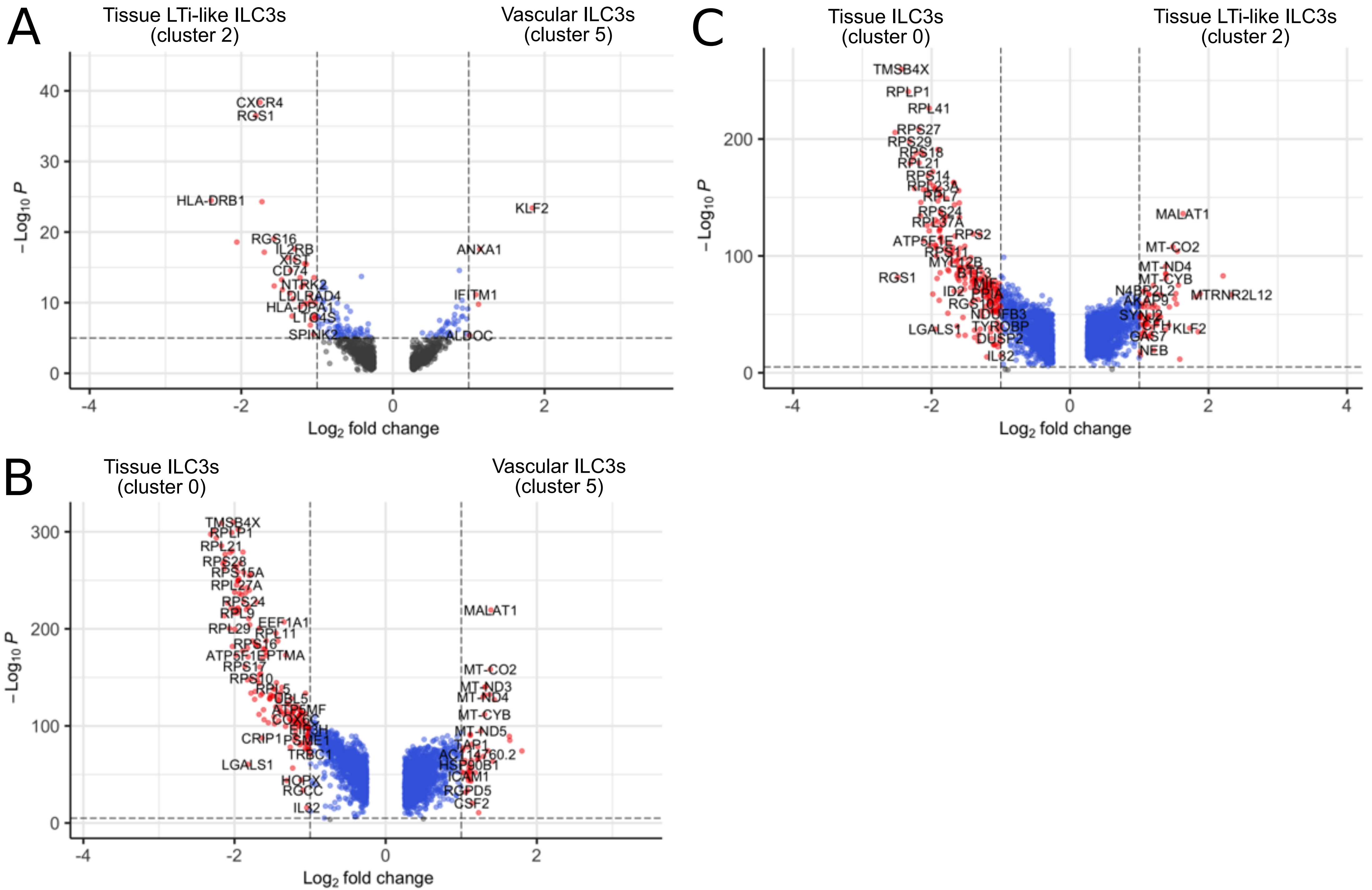
A
Tissue LTi-like ILC3s (cluster 2)
Vascular ILC3s (cluster 5)
CXCR4
RGS1
HLA-DRB1
RGS16
IL2RB
XIST
CD74
NTRK2
LDLRAD4
HLA-DPA1
LTC4S
SPINK2
KLF2
ANXA1
IFITM1
ALDOC
$-Log_{10}$ P
Log_2 fold change
B
Tissue ILC3s (cluster 0)
Vascular ILC3s (cluster 5)
TMSB4X
RPLP1
RPL21
RPS28
RPS15A
RPL27A
RPS24
RPL9
EEF1A1
RPL29
RPL11
RPS16
ATP5F1E
PTMA
RPS17
RPS10
RPL5
UBL5
ATP5MF
COX6C
CRIP1
PSME1
TRBC1
LGALS1
HOPX
RGCC
IL32
MALAT1
MT-CO2
MT-ND3
MT-ND4
MT-CYB
MT-ND5
TAP1
AC114760.2
HSP90B1
ICAM1
RGPD5
CSF2
C
Tissue ILC3s (cluster 0)
Tissue LTi-like ILC3s (cluster 2)
TMSB4X
RPLP1
RPL41
RPS27
RPS29
RPS18
RPL21
RPS14
RPL7
RPS24
RPL37A
RPS2
ATP5F1E
RPS11
MYL12B
BTF3
RGS1
ID2
MIF
PPIA
RGS10
NDUFB3
LGALS1
TYROBP
DUSP2
IL32
MALAT1
MT-CO2
MT-ND4
MT-CYB
N4BP2L2
AKAP9
MTRNR2L12
SYNJ2
CFH
KLF2
GAS7
NEB

Fig. S2. Differential gene expression between human ILC3 clusters from the lung of HSPC-engrafted MISTRG mice. (**A-C**) Volcano plots showing differentially expressed genes between the ILC3 clusters from Fig. 1A. Data are from one single-cell RNA-sequencing experiment with pooled lung cells from 10 MISTRG mice that were transplanted with human $CD34^+$ HSPCs from different donors.

Figure S3

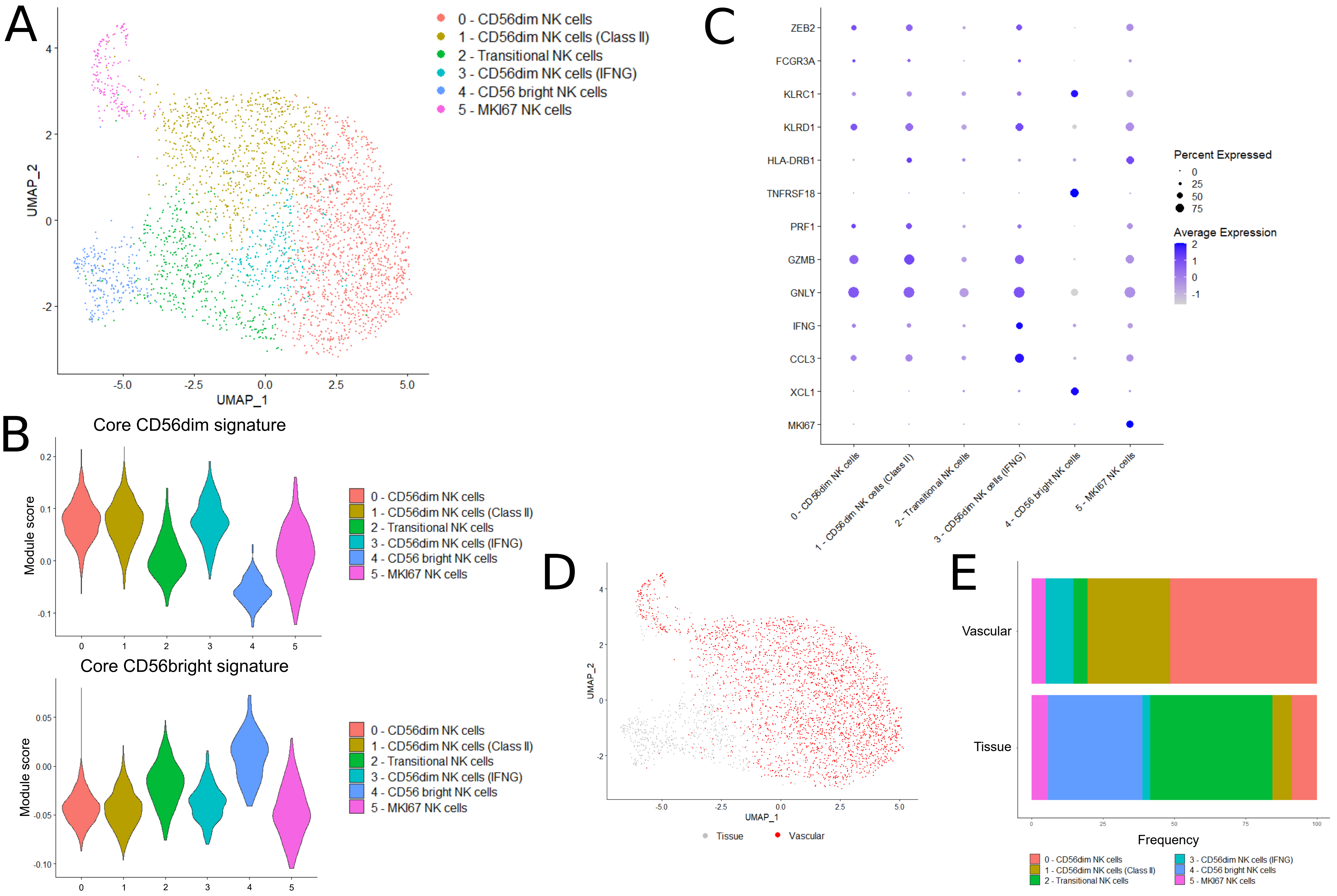

A
UMAP_1
UMAP_2
0 - CD56dim NK cells
1 - CD56dim NK cells (Class II)
2 - Transitional NK cells
3 - CD56dim NK cells (IFNG)
4 - CD56 bright NK cells
5 - MKI67 NK cells
B
Core CD56dim signature
Core CD56bright signature
Module score
C
ZEB2
FCGR3A
KLRC1
KLRD1
HLA-DRB1
TNFRSF18
PRF1
GZMB
GNLY
IFNG
CCL3
XCL1
MKI67
Percent Expressed
Average Expression
D
Tissue
Vascular
E
Vascular
Tissue
Frequency

Fig. S3. Diversity of human lung NK cells in the vascular and tissue compartment. (**A**) UMAP of human NK cell clusters found in the lung of HSPC-engrafted MITRG mice as revealed by single-cell RNA-sequencing. The UMAP shows 3,222 cells. (**B**) Gene similarity scores of the human NK cell clusters defined in (A) to those of $CD56^{dim}$ and $CD56^{bright}$ NK cells from humans. Core $CD56^{dim}$ and $CD56^{bright}$ NK cell gene signatures were obtained from (Dogra et al. 2020), see Supplemental Table 7. (**C**) Dot plot of selected genes in the human lung NK cell clusters from (A). (**D**) Vascular (red) and tissue (tissue) distribution of human NK cells from the lung of HSPC-engrafted MISTRG mice superimposed on the UMAP from (A). (**E**) Relative frequency of the human NK cells clusters in the vascular versus the tissue compartment of the MISTRG lung. Data are from one single-cell RNA-sequencing experiment with pooled lung cells from 9 MISTRG mice that were transplanted with human $CD34^{+}$ HSPCs from different donors.

Figure S4

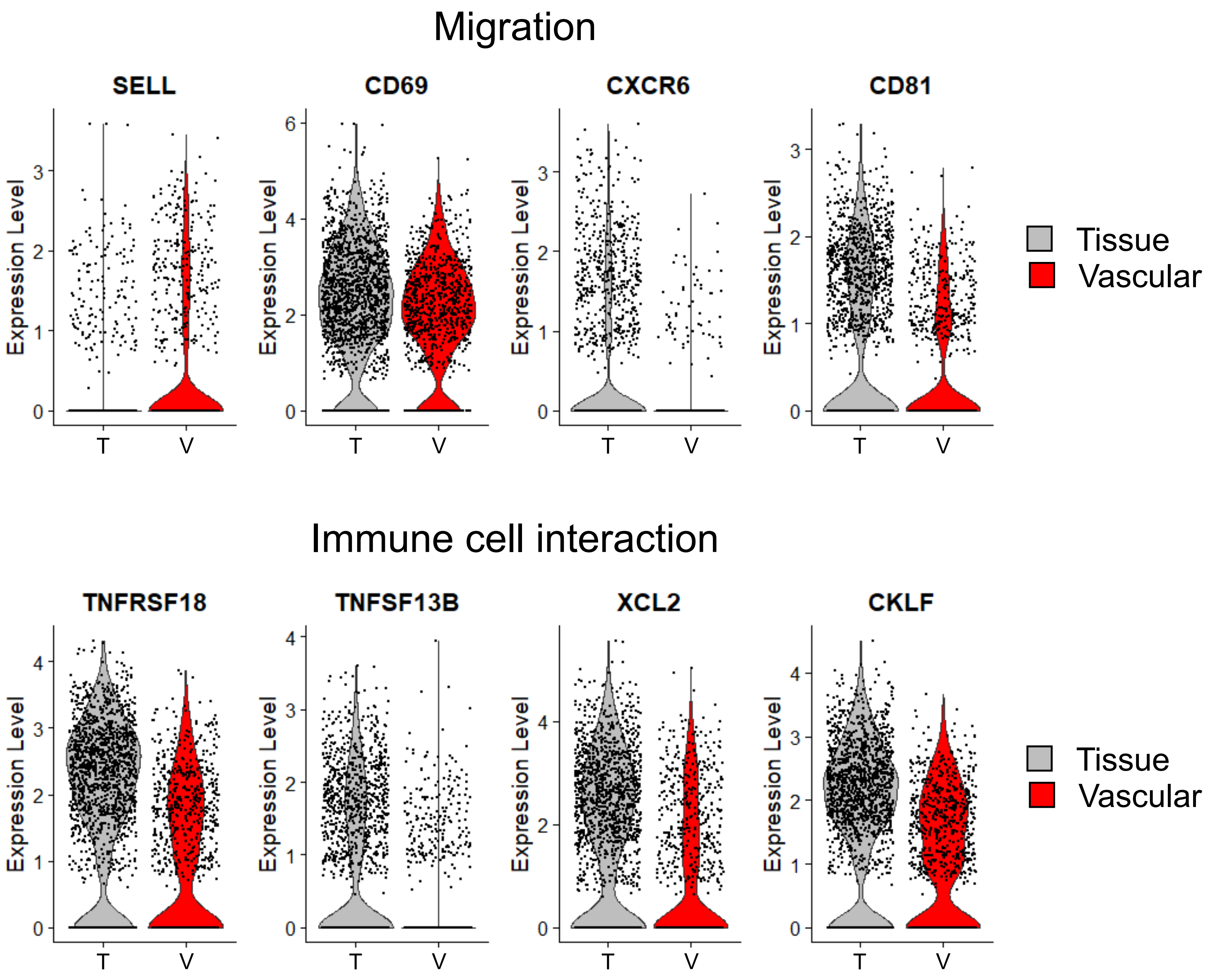
Migration
SELL
CD69
CXCR6
CD81
Expression Level
T
V
Tissue
Vascular
Immune cell interaction
TNFRSF18
TNFSF13B
XCL2
CKLF
Expression Level
T
V
Tissue
Vascular

Fig. S4. Selected differentially expressed genes in vascular versus tissue ILCs in the lung. Violin plots of selected genes that are differentially expressed between human ILCs in the lung vasculature (red) and human ILCs in the lung tissue (grey) from HSPC-engrafted MISTRG mice. Data are from one single-cell RNA-sequencing experiment with pooled lung cells from 10 MISTRG mice that were transplanted with human $CD34^+$ HSPCs from different donors.

Figure S5

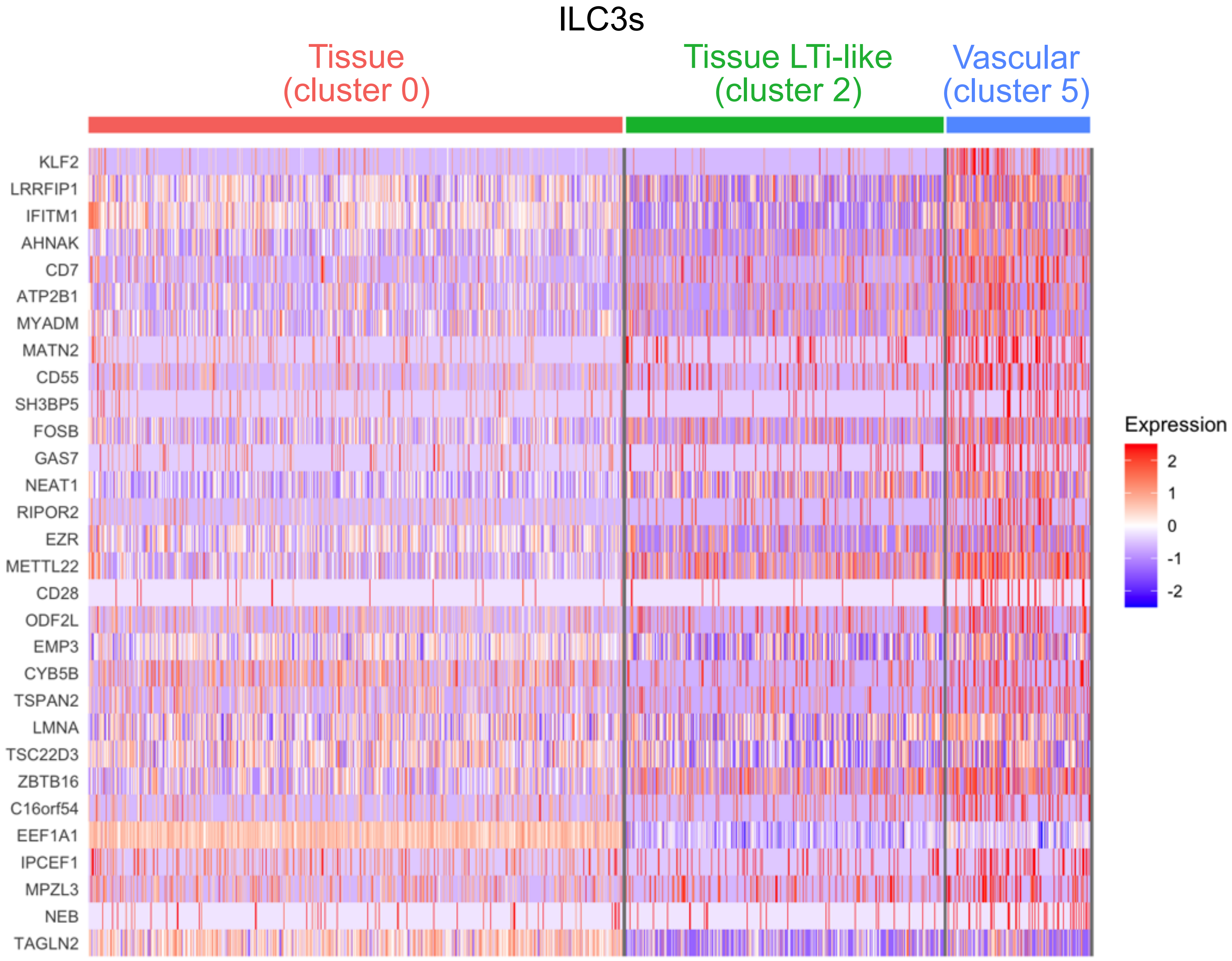
ILC3s
Tissue (cluster 0)
Tissue LTi-like (cluster 2)
Vascular (cluster 5)
KLF2
LRRFIP1
IFITM1
AHNAK
CD7
ATP2B1
MYADM
MATN2
CD55
SH3BP5
FOSB
GAS7
NEAT1
RIPOR2
EZR
METTL22
CD28
ODF2L
EMP3
CYB5B
TSPAN2
LMNA
TSC22D3
ZBTB16
C16orf54
EEF1A1
IPCEF1
MPZL3
NEB
TAGLN2
Expression
2
1
0
-1
-2

Fig. S5. Subset-specific transcriptional profiles of tissue-resident lung ILC2s and ILC3s. Heatmap of genes that are more highly expressed by vascular ILC3s (cluster 5) than tissue ILC3s (cluster 0 and cluster 2). Data are from one single-cell RNA-sequencing experiment with pooled lung cells from 10 MISTRG mice that were transplanted with human $CD34^+$ HSPCs from different donors.

Figure S6

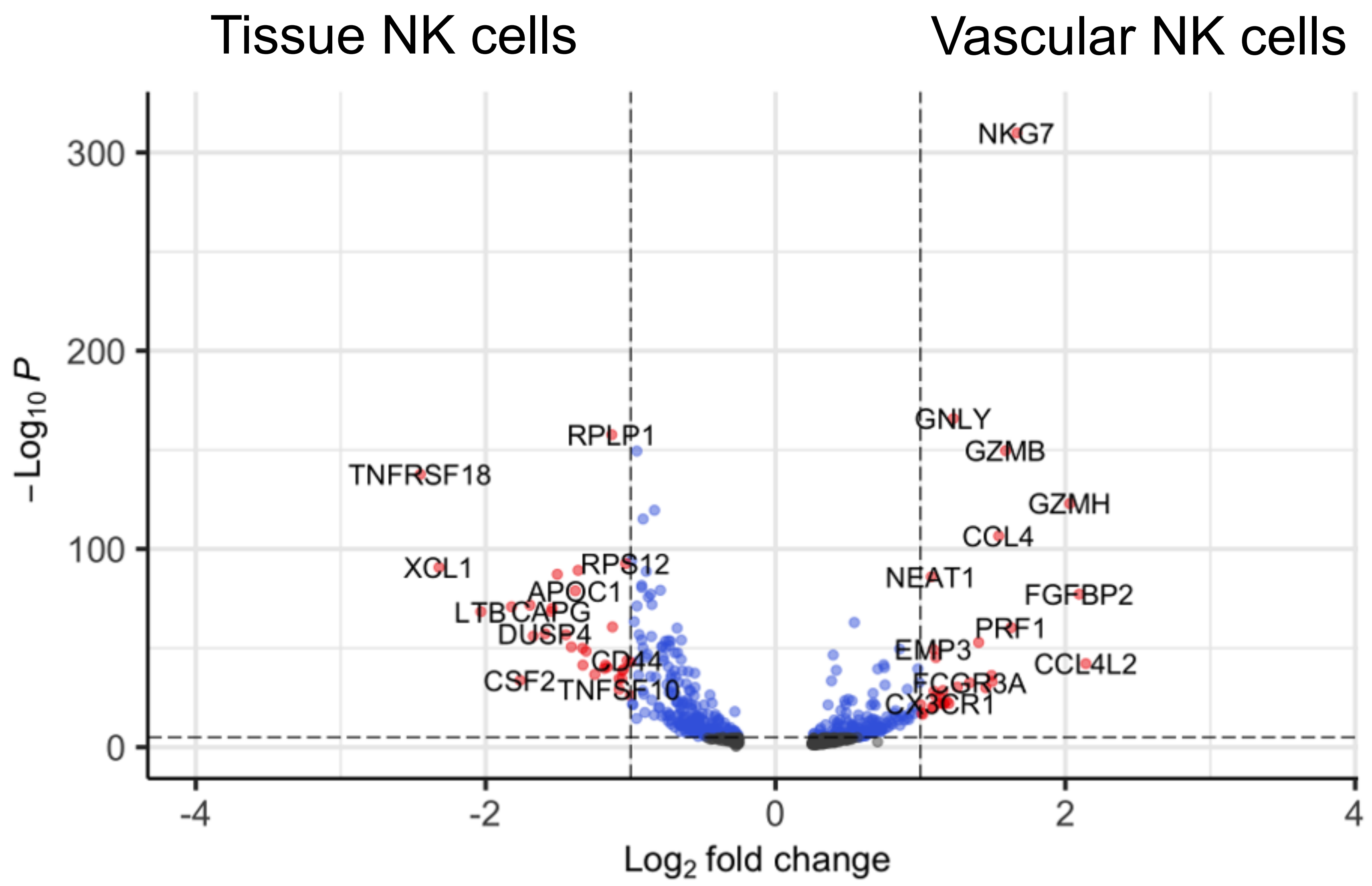

Tissue NK cells
Vascular NK cells
NKG7
GNLY
GZMB
GZMH
CCL4
NEAT1
FGFBP2
PRF1
EMP3
CCL4L2
FCGR3A
CX3CR1
RPLP1
TNFRSF18
XCL1
RPS12
APOC1
LTB
CAPG
DUSP4
CD44
CSF2
TNFSF10
300
200
100
0
-4
-2
0
2
4
−Log10 P
Log2 fold change

Fig. S6. Gene signatures of vascular and tissue NK cells from the lung of HSPC-engrafted MISTRG mice. Volcano plot shows genes upregulated in vascular or tissue NK cells from the lung of HSPC-engrafted MISTRG mice. Data are from one single-cell RNA-sequencing experiment with pooled lung cells from 9 MISTRG mice that were transplanted with human $CD34^+$ HSPCs from different donors.

Figure S7

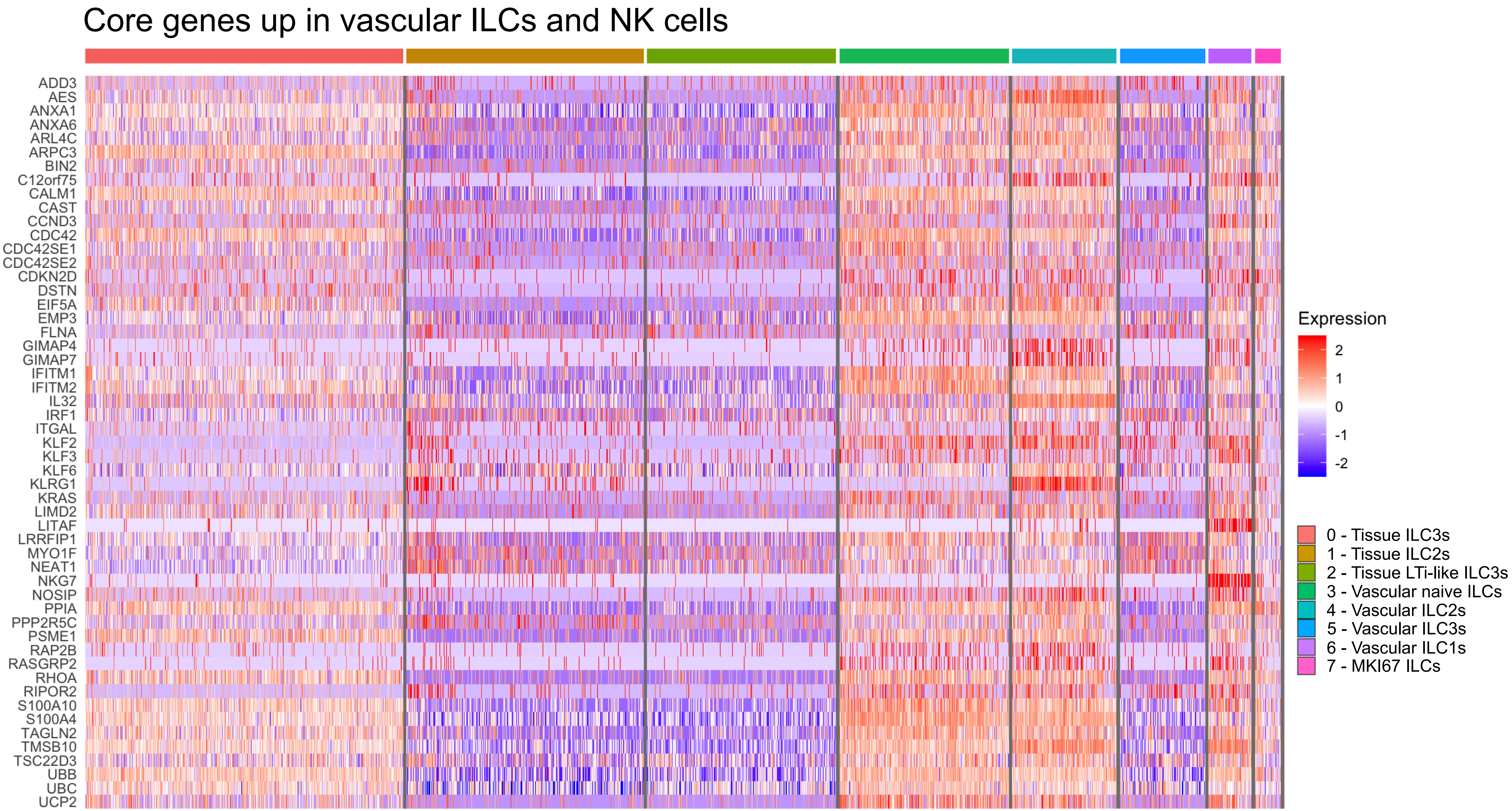

Core genes up in vascular ILCs and NK cells
ADD3
AES
ANXA1
ANXA6
ARL4C
ARPC3
BIN2
C12orf75
CALM1
CAST
CCND3
CDC42
CDC42SE1
CDC42SE2
CDKN2D
DSTN
EIF5A
EMP3
FLNA
GIMAP4
GIMAP7
IFITM1
IFITM2
IL32
IRF1
ITGAL
KLF2
KLF3
KLF6
KLRG1
KRAS
LIMD2
LITAF
LRRFIP1
MYO1F
NEAT1
NKG7
NOSIP
PPIA
PPP2R5C
PSME1
RAP2B
RASGRP2
RHOA
RIPOR2
S100A10
S100A4
TAGLN2
TMSB10
TSC22D3
UBB
UBC
UCP2
Expression
2
1
0
-1
-2
0 - Tissue ILC3s
1 - Tissue ILC2s
2 - Tissue LTi-like ILC3s
3 - Vascular naive ILCs
4 - Vascular ILC2s
5 - Vascular ILC3s
6 - Vascular ILC1s
7 - MKI67 ILCs

Fig. S7. Core gene signature of human ILCs and NK cells in the lung vasculature. Heatmap of genes that are upregulated in vascular human ILCs and NK cells from the lung of HSPC-engrafted MISTRG mice. Expression of core signature genes is shown in the ILC clusters from Fig. 1A.

Figure S8

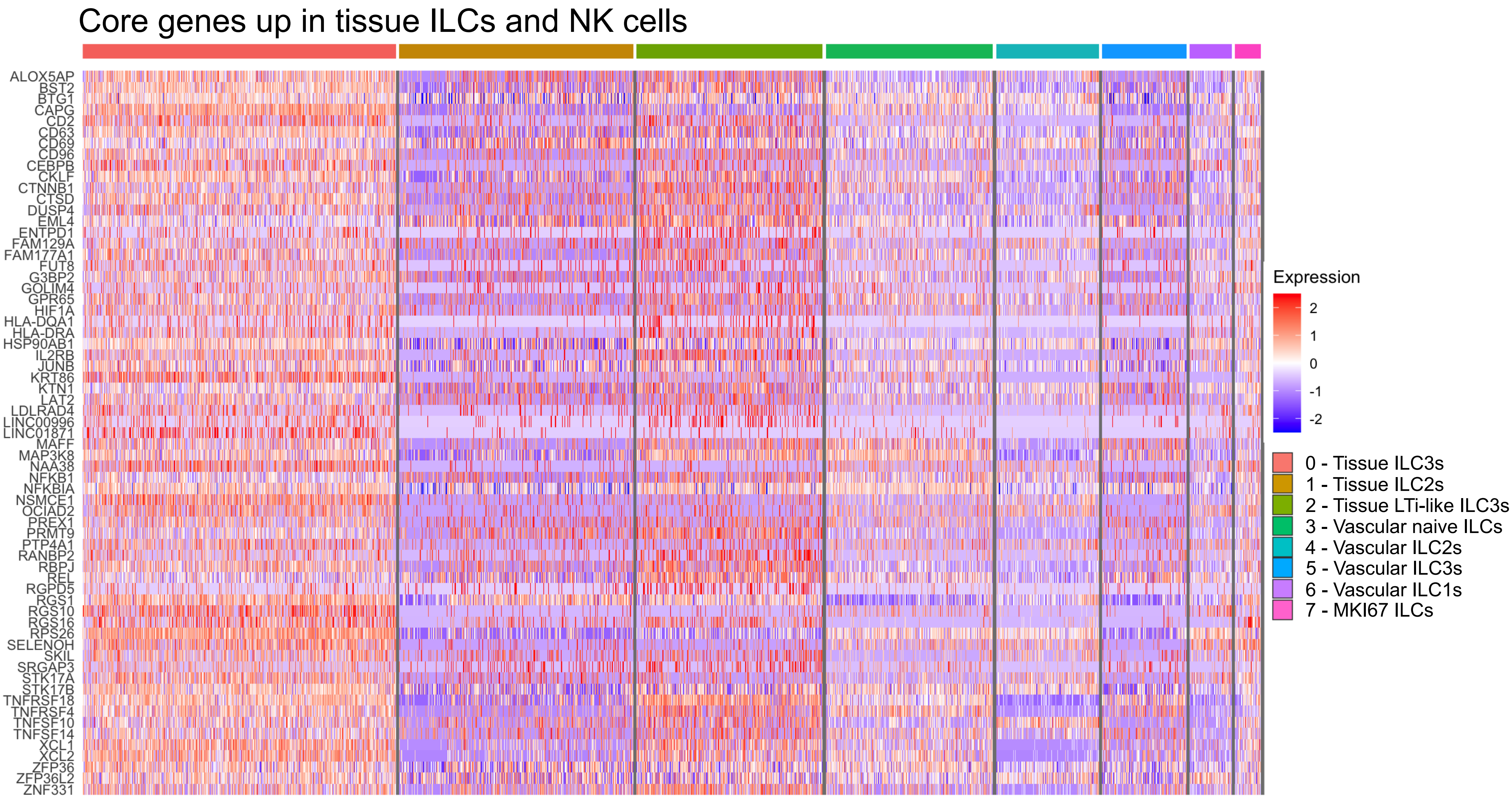
Core genes up in tissue ILCs and NK cells
ALOX5AP
BST2
BTG1
CAPG
CD2
CD63
CD69
CD96
CEBPB
CKLF
CTNNB1
CTSD
DUSP4
EML4
ENTPD1
FAM129A
FAM177A1
FUT8
G3BP2
GOLIM4
GPR65
HIF1A
HLA-DQA1
HLA-DRA
HSP90AB1
IL2RB
JUNB
KRT86
KTN1
LAT2
LDLRAD4
LINC00996
LINC01871
MAFF
MAP3K8
NAA38
NFKB1
NFKBIA
NSMCE1
OCIAD2
PREX1
PRMT9
PTP4A1
RANBP2
RBPJ
REL
RGPD5
RGS1
RGS10
RGS16
RPS26
SELENOH
SKIL
SRGAP3
STK17A
STK17B
TNFRSF18
TNFRSF4
TNFSF10
TNFSF14
XCL1
XCL2
ZFP36
ZFP36L2
ZNF331
Expression
2
1
0
-1
-2
0 - Tissue ILC3s
1 - Tissue ILC2s
2 - Tissue LTi-like ILC3s
3 - Vascular naive ILCs
4 - Vascular ILC2s
5 - Vascular ILC3s
6 - Vascular ILC1s
7 - MKI67 ILCs

Fig. S8. Core gene signature of human ILCs and NK cells in the lung tissue. Heatmap of genes that are upregulated in tissue human ILCs and NK cells from the lung of HSPC-engrafted MISTRG mice. Expression of core signature genes is shown in the ILC clusters from Fig. 1A.

Figure S9

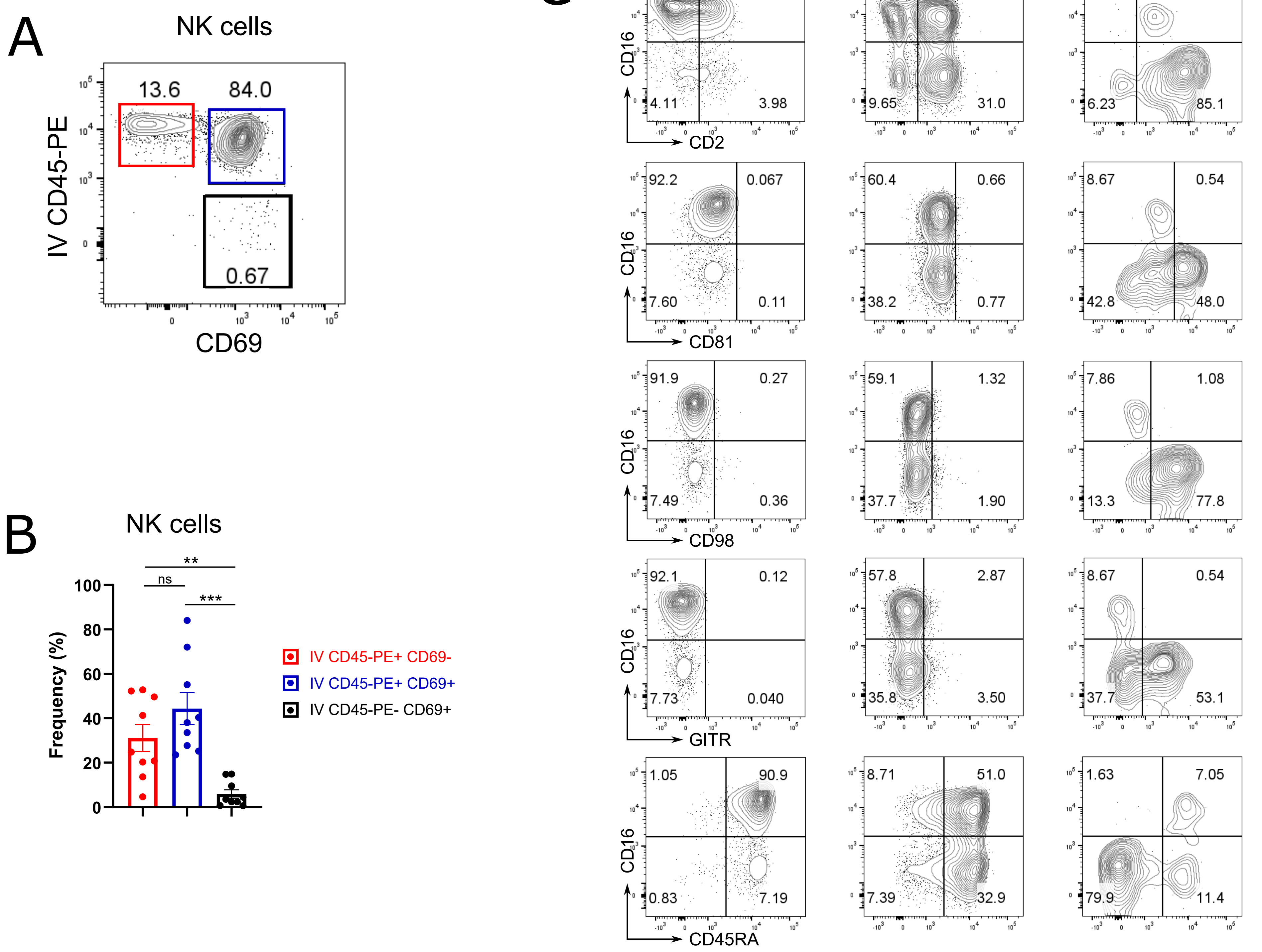
A
NK cells
13.6
84.0
0.67
IV CD45-PE
CD69
B
NK cells
Frequency (%)
ns
**
***
IV CD45-PE+ CD69-
IV CD45-PE+ CD69+
IV CD45-PE- CD69+
C
IV CD45-PE+ CD69-
IV CD45-PE+ CD69+
IV CD45-PE- CD69+
CD16
CD2
CD81
CD98
GITR
CD45RA
66.2
25.7
4.11
3.98
17.2
42.2
9.65
31.0
1.63
7.05
6.23
85.1
92.2
0.067
7.60
0.11
60.4
0.66
38.2
0.77
8.67
0.54
42.8
48.0
91.9
0.27
7.49
0.36
59.1
1.32
37.7
1.90
7.86
1.08
13.3
77.8
92.1
0.12
7.73
0.040
57.8
2.87
35.8
3.50
8.67
0.54
37.7
53.1
1.05
90.9
0.83
7.19
8.71
51.0
7.39
32.9
1.63
7.05
79.9
11.4

Fig. S9. Surface protein expression in tissue-adapted human lung NK cells. (**A**) Flow cytometry of lung NK cells from HSPC-engrafted MISTRG mice. Dot plots show IV CD45-PE labeling and CD69 surface expression. NK cells were gated as $CD45^{+}Lin^{-}CD3^{-}TCRab^{-}CD127^{-}CD94^{+}$ cells. (**B**) Frequency of IV $CD45\text{-}PE^{+}CD69^{-}$, IV $CD45\text{-}PE^{+}CD69^{+}$, and IV $CD45\text{-}PE^{-}CD69^{+}$ cells among lung NK cells. Error bars indicate SEM. n.s., not significant; **, P<0.01; ***, P<0.001 by one-way ANOVA with post hoc testing. (**C**) Surface expression of CD2, CD81, CD98, GITR, and CD45RA on human lung NK cells according to IV $CD45\text{-}PE^{+}CD69^{-}$, IV $CD45\text{-}PE^{+}CD69^{+}$, and IV $CD45\text{-}PE^{-}CD69^{+}$ subsets. Data are representative of at least 3 independent experiments (n=7-9 mice).

Figure S10

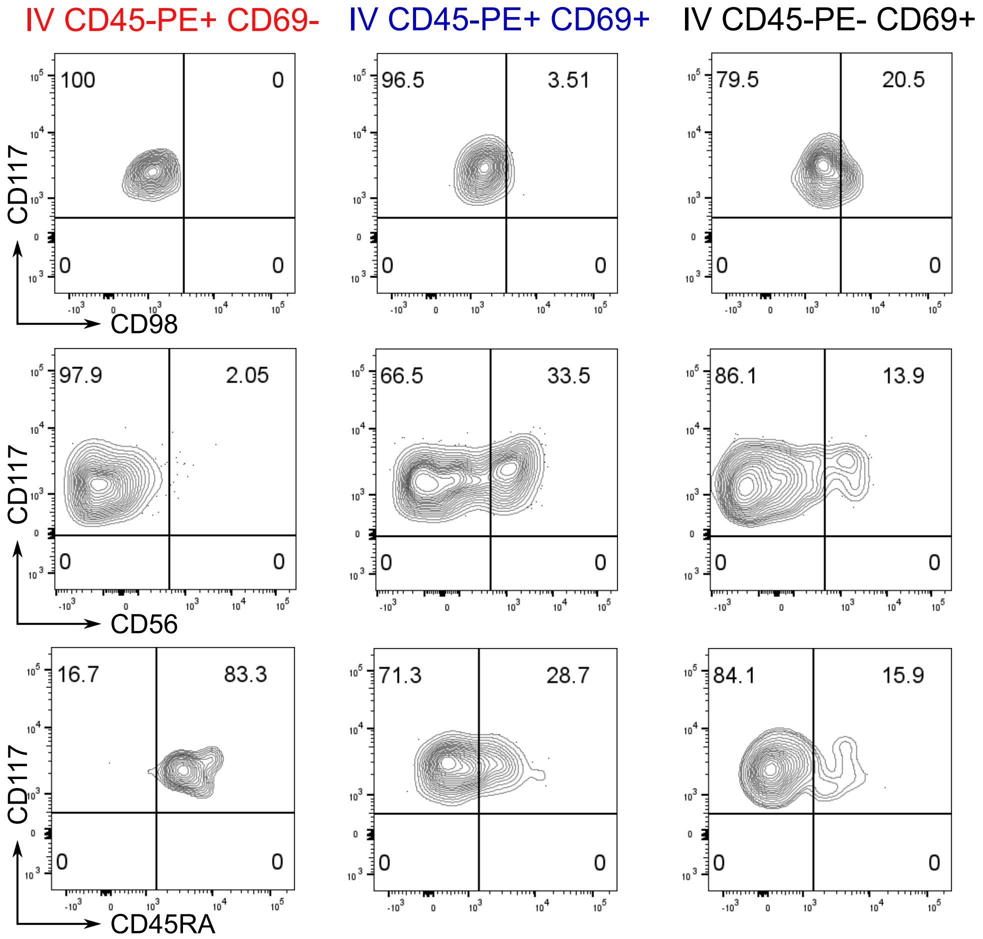

IV CD45-PE+ CD69-
IV CD45-PE+ CD69+
IV CD45-PE- CD69+
CD117
CD98
CD56
CD45RA
100
0
0
0
96.5
3.51
0
0
79.5
20.5
0
0
97.9
2.05
0
0
66.5
33.5
0
0
86.1
13.9
0
0
16.7
83.3
0
0
71.3
28.7
0
0
84.1
15.9
0
0

Fig. S10. Surface protein expression in human vascular and tissue ILC3s in the lung. Surface expression of CD98, CD56, and CD45RA on human lung ILC3s according to IV CD45-PE^{+}CD69, IV CD45-PE^{+}CD69^{+}, and IV CD45-PE^{-}CD69^{+} subsets. Data are representative of 4-5 independent experiments (n=8-10 mice).
